# Primary restriction of S‐RNase cytotoxicity by a stepwise ubiquitination and degradation pathway in *Petunia* *hybrida*

**DOI:** 10.1111/nph.17438

**Published:** 2021-05-30

**Authors:** Hong Zhao, Yanzhai Song, Junhui Li, Yue Zhang, Huaqiu Huang, Qun Li, Yu’e Zhang, Yongbiao Xue

**Affiliations:** ^1^ State Key Laboratory of Plant Cell and Chromosome Engineering Institute of Genetics and Developmental Biology, and The Innovation Academy of Seed Design Chinese Academy of Sciences Beijing 100101 China; ^2^ University of Chinese Academy of Sciences Beijing 100049 China; ^3^ Beijing Institute of Genomics Chinese Academy of Sciences and National Centre for Bioinformation Beijing 100101 China; ^4^ Jiangsu Co‐Innovation Centre for Modern Production Technology of Grain Crops Yangzhou University Yangzhou 225009 China

**Keywords:** *Petunia* *hybrida*, S‐RNase, self‐incompatibility, SLF, ubiquitination

## Abstract

- In self‐incompatible *Petunia* species, the pistil S‐RNase acts as cytotoxin to inhibit self‐pollination but is polyubiquitinated by the pollen‐specific nonself *S*‐locus F‐box (SLF) proteins and subsequently degraded by the ubiquitin‐proteasome system (UPS), allowing cross‐pollination. However, it remains unclear how S‐RNase is restricted by the UPS.
- Using biochemical analyses, we first show that *Petunia hybrida* S_3_‐RNase is largely ubiquitinated by K48‐linked polyubiquitin chains at three regions, R I, R II and R III. R I is ubiquitinated in unpollinated, self‐pollinated and cross‐pollinated pistils, indicating its occurrence before PhS_3_‐RNase uptake into pollen tubes, whereas R II and R III are exclusively ubiquitinated in cross‐pollinated pistils.
- Transgenic analyses showed that removal of R II ubiquitination resulted in significantly reduced seed sets from cross‐pollination and that of R I and R III to a lesser extent, indicating their increased cytotoxicity. Consistent with this, the mutated R II of PhS_3_‐RNase resulted in a marked reduction of its degradation, whereas that of R I and R III resulted in less reduction.
- Taken together, we demonstrate that PhS_3_‐RNase R II functions as a major ubiquitination region for its destruction and R I and R III as minor ones, revealing that its cytotoxicity is primarily restricted by a stepwise UPS mechanism for cross‐pollination in *P. hybrida*.

## Introduction

Self‐incompatibility (SI), an inability of a fertile seed plant to produce a zygote after self‐pollination, represents a reproductive barrier adopted by nearly 40% of flowering plant species to prevent self‐fertilisation and to promote outcrossing (De Nettancourt, 2001). In many species, SI is usually controlled by a single multiallelic *S*‐locus encoding both male and female *S*‐determinants (De Nettancourt, 2001; Takayama & Isogai, 2005; Franklin‐Tong, 2008; Zhang *et al*., 2009). Their molecular interaction confers the pistil with the ability to distinguish between genetically related self‐pollen and nonself‐pollen. In general, SI can be classified into self‐recognition and nonself‐recognition systems based on their distinct molecular mechanisms (Fujii *et al*., 2016). In the self‐recognition system of Papaveraceae and Brassicaceae, self‐pollen rejection occurs as a specific interaction between the *S*‐determinants from the same *S* haplotype. In *Papaver rhoeas*, the female *S*‐determinant Prs S (*P*. *rhoeas* stigmatic S) interacts with its cognate Prp S (*P*. *rhoeas* pollen S) to stimulate a signalling cascade leading to programmed cell death (PCD) of self‐pollen (Foote *et al*., 1994; Thomas & Franklin‐Tong, 2004; Wheeler *et al*., 2009; Wilkins *et al*., 2014). In Brassicaceae, the SI response is initiated by the specific interaction of the stigma *S*‐locus receptor kinase (SRK) and its cognate pollen‐coat‐localised ligand *S*‐locus cysteine‐rich protein (SCR/SP11), triggering a phosphorylation‐mediated signalling pathway and resulting in the destruction of factors indispensable for pollen compatibility by the UPS (Schopfer *et al*., 1999; Suzuki *et al*., 1999; Takasaki *et al*., 2000; Takayama *et al*., 2000; Kakita *et al*., 2007; Samuel *et al*., 2008, 2009; Ma *et al*., 2016). S‐RNase‐based SI, also termed as Solanaceae‐type SI, is a well studied nonself‐recognition system widely present in Solanaceae, Plantaginaceae, Rosaceae and Rutaceae (Anderson *et al*., 1986; McClure *et al*., 1989; Sassa *et al*., 1996; Xue *et al*., 1996; Lai *et al*., 2002; Ushijima *et al*., 2003; Sijacic *et al*., 2004; Liang *et al*., 2020). In self‐incompatible *Antirrhinum* and *Petunia* species, the pistil *S*‐determinant S‐RNase serving as a cytotoxin can be recognised and ubiquitinated by multiple pollen *S*‐determinant SLFs to form functional SCF ubiquitin ligases in a collaborative nonself‐recognition manner, therefore restricting cytotoxicity of nonself S‐RNases and resulting in cross‐pollination (Qiao *et al*., 2004b; Hua & Kao, 2006, 2008; Hua *et al*., 2008; Zhang *et al*., 2009; Kubo *et al*., 2010; Liu *et al*., 2014).

However, it remains largely unclear how S‐RNases are specifically regulated in the nonself‐recognition system. Currently, two models, the S‐RNase degradation model and the S‐RNase compartmentalisation model, have been proposed to explain how S‐RNase cytotoxicity is restricted for cross‐pollination (Qiao *et al*., 2004b; Goldraij *et al*., 2006; McClure, 2009, 2011; Liu *et al*., 2014). In the degradation model proposed in *Petunia hybrida*, both self and nonself S‐RNases taken up by pollen tubes are mainly localised in the cytosol, where they are further recognised by SLFs. Entani *et al*. (2014) showed that SCF^SLF^ complexes can specifically polyubiquitinate nonself S‐RNases rather than self S‐RNases *in vitro* in *P*. *hybrida*, providing evidence for S‐RNase ubiquitination by cross‐pollen. Alternatively, in self‐pollen tubes, the binding of self S‐RNase and SLF leads to the formation of the nonfunctional SCF^SLF^ complex, therefore resulting in the survival of self S‐RNase to inhibit pollen‐tube growth. Together with the discoveries of SCF^SLF^ complex components such as SLF‐interacting SKP1‐like 1 (SSK1) and Cullin1 in species from Solanaceae, Plantaginaceae and Rosaceae (Huang *et al*., 2006; Zhao *et al*., 2010; Xu *et al*., 2013; Entani *et al*., 2014; Li & Chetelat, 2014), the degradation model appears to function in several species of flowering plants that possess S‐RNase‐based SI. In *Nicotiana* species, Goldraij *et al*. (2006) proposed that the majority of self S‐RNases and nonself S‐RNases would be sequestered in vacuole‐like structures once imported into pollen tubes. Subsequently, self‐recognition between SLFs and a small fraction of S‐RNases localised in the cytosol would break the structures, releasing S‐RNases in a late stage of self‐pollination, and triggering the SI response. By contrast, nonself‐recognition could stabilise S‐RNases and maintain their sequestration. Most previous studies have shown that S‐RNase degradation rather than compartmentalisation acts as the major strategy to restrict S‐RNase cytotoxicity in *P*. *hybrida* (Liu *et al*., 2014). Nevertheless, little information is known about the linkage type of the polyubiquitin chains and the specific residue of S‐RNase ubiquitinated by nonself SCF^SLF^ complexes in cross‐pollen tubes.

To address these questions, in this study, we first established an *in vivo* assay to examine the polyubiquitination of PhS_3_‐RNase in cross‐pollinated pistils and, together with *in vitro* ubiquitination analyses, we found that PhS_3_‐RNase was mainly ubiquitinated by K48‐linked polyubiquitin chains in three regions named R I, R II and R III. Among them, R I ubiquitination occurred before PhS_3_‐RNase entry into pollen tubes and is likely to be mediated by an unknown E3 ligase, whereas R II and III were specifically ubiquitinated by SCF^SLF^. Second, the ubiquitination removal of those three regions had little effect on the physicochemical properties of PhS_3_‐RNase, but negatively impacted their functions in cross‐pollen tubes. The transgene with a mutated R II led to a significant reduction of seed sets from cross‐pollination, whereas in mutated R I and R III seeds sets were reduced to a much lesser extent in *P*. *hybrida*, showing that R II ubiquitination of PhS_3_‐RNase played a major role in its destruction and cytotoxicity restriction, whereas R I and III had minor roles. Furthermore, the ubiquitination removal of all three regions did not completely inhibit PhS_3_‐RNase degradation and cross seed sets, suggesting that UPS was not the exclusive mechanism to restrict S‐RNase cytotoxicity. Taken together, our results demonstrated a stepwise UPS mechanism for primary restriction of S‐RNase cytotoxicity during cross‐pollination in *P*. *hybrida*, providing novel mechanistic insight into a dynamic regulation of S‐RNases.

## Materials and Methods

### Plant materials

Self‐incompatible wild‐type lines of *PhS_3_S_3_*, *PhS_V_S_V_* and *PhS_3_S_3L_* have been previously described (Robbins *et al*., 2000; Sims & Ordanic, 2001). The transgenic plants *PhS_3_S_3L_*/*PhS_3L_SLF1*‐*FLAG* were constructed by transforming *PhS_3_S_3L_* with *pBI101*‐*PhS_3_A*‐*SLF::PhS_3L_SLF1*‐*FLAG*. *PhS_3_A*‐*SLF* is a native promotor used for *PhSLFs* expression as previously described (Qiao *et al*., 2004a; Liu *et al*., 2014).

### Ti plasmid construction and transgenic plant generation

*PhS_3_‐RNase* cDNA and its *FLAG*‐tagged form were amplified by primers listed in Supporting Information Table S1 to introduce *Xho*I and *Sac*I restriction sites at their 5′ and 3′ ends, respectively. *PhS_3_‐RNase* point mutations were generated by polymerase chain reaction (PCR) using site‐directed mutagenesis primers listed in Table S1. Ti plasmid constructs were separately electroporated into *Agrobacterium tumefaciens* strain LBA4404 and introduced into *PhS_3_S_3L_* using the leaf disc transformation method as described previously (Lee *et al*., 1994; Qiao *et al*., 2004a).

### Protein structure prediction and electrostatic potential analysis

PhS_3_‐RNase protein structure was modelled using the I‐TASSER server (http://zhanglab.ccmb.med.umich.edu/I‐TASSER/) according to its instructions (Yang *et al*., 2015; Li *et al*., 2017). The first model generated by iterative simulations was selected for further analysis based on model quality evaluation using the Vadar v.1.8 program (http://vadar.wishartlab.com; Willard *et al*., 2003) and the prosa‐web program (https://prosa.services.came.sbg.ac.at/prosa.php; Wiederstein & Sippl, 2007). Structures of point‐mutated S‐RNases were generated by mutagenesis in PyMol, and electrostatic potential analyses of PhS_3_‐RNase and its point‐mutated structures were performed using plug‐in APBS tools as previously described (Baker *et al*., 2001; Li *et al*., 2017).

### Quantitative (q) RT‐PCR analysis

Total RNAs were separately isolated from pistils derived from *PhS_3_S_3L_*/*PhS_3_*‐*RNase*‐ or *PhS_3_*‐*RNase* (*Mutant*) (*M*)‐(*FLAG*) using TRIzol reagent (Ambion) according to the manufacturer’s instructions. cDNA was subsequently synthesised using cDNA synthesis supermix (AU311‐02; Transgen, Beijing, China). qRT‐PCR reaction mixes were prepared according to manufacturer’s guidelines of ChamQ™ Universal SYBR qPCR Master Mix (Q711‐02/03, Vazyme, Nanjing, China). Relevant primer sequences are listed in Table S1. qRT‐PCR assays were performed using the CFX96™ Real‐Time System (Bio‐Rad). *Petunia* *hybrida 18S rRNA* gene transcripts were used as an internal control. The data were analysed following the method of Livak ($2^{‐\Delta \Delta C_{t}}$) (Livak & Schmittgen, 2001).

### Mass spectrometry analysis for ubiquitination sites

*PhS_3_S_3L_* plants were self‐pollinated or cross‐pollinated with *PhS_3_S_3L_*/*PhS_3L_SLF1*. Then the pollinated pistils collected after 2, 6, 12 and 24 h, respectively, were mixed up, minced and lysed in buffer containing 7 M urea, 2 M thiourea and 0.1% CHAPS, and followed by 5‐min ultrasonication on ice. Samples of unpollinated pistils were prepared as controls. The lysate was centrifuged at 14 000 ***g*** for 10 min at 4°C and the supernatant was reduced with 10 mM DTT for 1 h at 56°C, and subsequently alkylated with sufficient iodoacetamide for 1 h at room temperature in the dark (Udeshi *et al*., 2013b). Then the supernatant containing 10 mg protein was digested with Trypsin Gold (Promega) at enzyme : substrate ratio 1 : 50 at 37°C for 16 h. Peptides were desalted with C18 cartridge and dried by vacuum centrifugation (Udeshi *et al*., 2013b), and then resuspended in MOPS IAP buffer (50 mM MOPS, 10 mM KH_2_PO_4_, 50 mM NaCl, pH 7.0) and centrifuged for 5 min at 12 000 ***g***. The supernatants were incubated with anti‐Ubiquitin Remnant Motif (K‐ε‐GG) beads (CST #5562; Cell Signaling Technology, Beverly, MA, USA) for 2.5 h at 4°C and centrifuged for 30 s at 3000 ***g*** at 4°C. Beads were washed in MOPS IAP buffer, then in water, before elution of the peptides with 0.15% TFA (Udeshi *et al*., 2013a). Then desalted by peptide desalting spin columns (89852; Thermo Fisher, Waltham, MA, USA) before LC‐MS/MS analysis using an Orbitrap Fusion mass spectrometer (Thermo Fisher). The resulting spectra from each fraction were searched separately against PhS_3_‐RNase amino acid sequences using the Maxquant search engines. Precursor quantification based on intensity was used for label‐free quantification.

### S‐RNase activity assay

The coding sequences of *PhS_3_‐RNase* (without signal peptide) and *PhS_3_*‐*RNase* (*M*) were separately cloned into the *pCold*‐*TF* vector (TaKaRa, Kusatsu, Japan). Relevant primer sequences used are listed in Table S1. Trigger Factor (TF) is a 48 kDa soluble tag located at the N‐terminus of His. The His‐fused proteins were expressed in *Escherichia coli* Trans BL21 (DE3) plysS (Transgen) at 16°C for 24 h at 180 rpm and purified using Ni Sepharose 6 Fast Flow beads (10249123; GE Healthcare, Waukesha, WI, USA) according to the manufacturer’s instructions. Protein concentration was determined by Bradford protein assays. The relative fluorescence units (RFU) of the recombinant proteins were monitored according to the manufacturer’s instructions of RNase Alert Lab Test Kit (Ambion) using Synergy 2 (Biotek, Winooski, VT, USA).

### Ubiquitination assay and immunoblotting

The SCF^SLF‐FLAG^ complex attached to anti‐FLAG M2 affinity gel (Sigma‐Aldrich) was purified from *PhS_3_S_3L_*/*PhS_3L_SLF1*‐*FLAG* pollen tubes as described (Li *et al*., 2017), with that from *PhS_3_S_3L_* as a negative control. PhS_3_‐RNase was purified from *PhS_3_S_3_* pistils through Fast protein liquid chromatography (FPLC) as described previously (Entani *et al*., 2014; Li *et al*., 2017), recombinant His‐PhS_3_‐RNase and ‐PhS_3_‐RNase (M) were used separately as a substrate for the ubiquitination reactions (Li *et al*., 2017). For immunoblotting, PVDF membranes (Millipore, Burlington, MA, USA) were probed with primary antibodies, including mouse monoclonal anti‐PhS_3_‐RNase, anti‐ubiquitin (Abgent, San Diego, CA, USA), and anti‐His (Sigma) antibodies at a 1 : 2000 dilution for western blot detection. ImageJ software was used to quantify the immunosignal of the ubiquitinated proteins. K48‐ or K63‐linkage specific polyubiquitin rabbit mAb (Cell Signaling) at a 1 : 1000 dilution was used to analyse the linkage type of PhS_3_‐RNase ubiquitination.

### Subcellular fractionation and pulse–chase assays

For the subcellular fractionation assay, mature pollen grains of *PhS_V_S_V_* were cultured, germinated and collected as described previously (Liu *et al*., 2014). Style extracts of transgenic pistils containing PhS_3_‐RNase‐FLAG or PhS_3_‐RNase (M)‐FLAG were separately used to incubate the germinated pollen tubes. Then the pollen tubes were harvested for fractionation and equal amounts of protein samples derived from each centrifugation step were applied to immunoblots, as described previously (Liu *et al*., 2014). For the pulse–chase assay, the germinated pollen tubes of *PhS_V_S_V_* or *PhS_3_S_3_* were incubated separately for 1 h with style extracts from the transgenic pistils containing PhS_3_‐RNase‐FLAG or PhS_3_‐RNase (M)‐FLAG that were collected and rinsed and allowed to grow further in fresh medium with or without MG132 for *c*. 5 h. Equal amounts of pollen‐tube samples were loaded and detected by immunoblotting using anti‐FLAG antibodies (Sigma).

### Cell‐free degradation and ubiquitination assays

Total proteins of germinated *PhS_V_S_V_* pollen tubes were extracted on ice using cell‐free degradation buffer containing 25 mM Tris‐HCl (pH 7.5), 10 mM NaCl, 10 mM MgCl_2_, 1 mM DTT, 10 mM ATP and 1 mM PMSF. Then, equal amounts of extracts were applied to react with recombinant SUMO‐His‐PhS_3_‐RNase or its ‐PhS_3_‐RNase (M) with or without MG132 at 30°C. Equal amounts of samples were taken out at indicated time points for immunoblots. ImageJ software was used to quantify the immunosignals. The SUMO‐His‐tagged fusion proteins were generated as follows. The coding sequences of *PhS_3_‐RNase* (without signal peptide) and *PhS_3_‐RNase* (*M*) were separately cloned into engineered *pET*‐*30a* (Novagen, Madison, WI, USA) containing N‐terminal SUMO tag to produce SUMO‐His‐tagged proteins. Relevant primer sequences are listed in Table S1. The fusion proteins were, respectively, expressed in *E*. *coli* Trans BL21 (DE3) plysS (Transgen) at 16°C for 24 h, and then purified using Ni Sepharose 6 Fast Flow beads. For the cell‐free ubiquitination assays, equal amounts of recombinant His‐PhS_3_‐RNase and PhS_3_‐RNase (M) were separately incubated with *PhS_3_S_3_* pollen‐tube extracts using ubiquitin reaction buffer as described previously (Hua & Kao, 2006). Then the His‐fused substrates and their ubiquitinated forms were purified from the reaction products through Ni Sepharose 6 Fast Flow beads for immunoblots as described above.

### Pull‐down assay

The coding sequences of *PhS_3_*‐*RNase* (without signal peptide) and *PhS_3_‐RNase* (*M*) were cloned separately into *pMAl*‐*c2x* (Novagen) to generate MBP‐tagged fusion proteins. The full length of *PhS_3L_SLF1* was cloned into engineered *pET*‐*30a* (Novagen) described above to produce SUMO‐His‐PhS_3L_SLF1. Relevant primer sequences used are listed in Table S1. All the recombinant proteins were induced overnight at 16°C at 180 rpm, with MBP‐tagged proteins expressed in *E. coli* Trans BL21 (DE3) plysS cells described above and SUMO‐His‐PhS_3L_SLF1 in *E. coli* Trans BL21 (DE3) (Transgen). Cells were collected and resuspended using binding buffer (20 mM Tris‐HCl (pH 8.0), 200 mM NaCl, 1 mM DTT and 1 mM EDTA (pH 8.0)) for ultrasonication on ice. Then equal lysates containing SUMO‐His‐PhS_3L_SLF1 were incubated with the same amount of lysates containing MBP or MBP‐tagged fusion protein (PhS_3_‐RNase or its six mutant forms), respectively, for 2 h at 4°C. The mixed lysates were subsequently immobilised on Dextrin Sepharose High Performance medium (GE HealthCare, 10284602) following the manufacturer’s instructions and eluted by binding buffer supplemented with 10 mM maltose for immunoblots using anti‐MBP (NEB) and anti‐His (Sigma) antibodies.

### Bimolecular fluorescence complementation (BiFC) and split firefly luciferase complementation (SFLC) assays

For the BiFC assay, the coding sequences of *PhS_3_*‐*RNase* (without signal peptide) and *PhS_3_‐RNase* (*M*) were amplified separately and inserted into *pSY*‐*735*‐*35S*‐*cYFP*‐*HA* and the full‐length cDNA of *PhS_3L_SLF1* was cloned into *pSY*‐*736*‐*35S*‐*nYFP*‐*EE* as described previously (Li *et al*., 2018). Relevant primer sequences used are listed in Table S1. Different construct combinations (e.g. *nYFP*‐*PhS_3L_SLF1* and *cYFP*‐*PhS_3_*‐*RNase*), together with the *p19* silencing plasmid, were cotransfected into tobacco leaf epidermal cells using *Agrobacterium* (GV3101)‐mediated infiltration to generate fusion proteins (e.g. nYFP‐PhS_3L_SLF1 and cYFP‐PhS_3_‐RNase) for their interaction test. After culture for another 48 h in the dark, a portion of the injected leaf was cut off and examined by confocal microscope (Zeiss LSM710) to capture the YFP signal. For the SFLC assay, *PhS_3L_SLF1* (or *PhS_3_SLF1*) and *PhS_3_*‐*RNase* (or its mutant forms) were cloned into *pCAMBIA1300*‐*35S*‐*HA*‐*nLUC*‐*RBS* and *pCAMBIA1300*‐*35S*‐*cLUC*‐*RBS* vectors, respectively, as described previously (Liu *et al*., 2018). At 48 h post‐injection, 1 mM luciferin was sprayed on the injected leaves and the LUC signals were captured using a cooled CCD imaging system (LB985; Berthold, Bad Wildbad, Germany).

### Aniline blue staining of pollen tubes within pistils

Pollinated pistils were chemically fixed and treated for observation as described (Liu *et al*., 2014).

## Results

### S‐RNase polyubiquitination mainly occurs through K48 linkages at three conserved spatial regions among S‐RNases

**Fig. 1:**
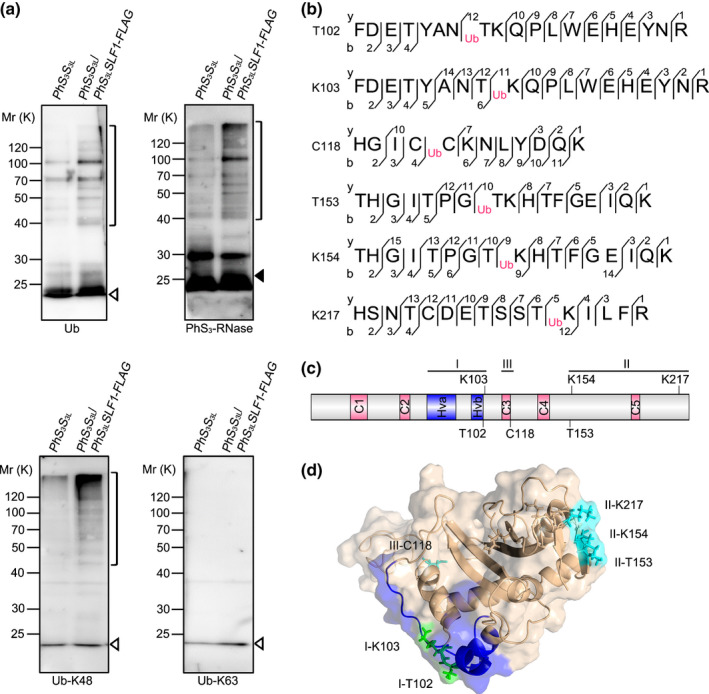
Six amino acid residues of *Petunia hybrida* S_3_‐RNase are ubiquitinated by K48‐linked polyubiquitin chains through SCF^PhS3LSLF1^. (a) Immunoblot detection of *in vitro* ubiquitinated products of PhS_3_‐RNase by PhS_3L_SLF1. The pollen genotypes and the transgene are indicated on top and *PhS_3_S_3L_* used as a negative control. Brackets indicate polyubiquitinated PhS_3_‐RNases. Open and closed arrowheads indicate ubiquitin and unubiquitinated PhS_3_‐RNase monomers, respectively. Antibodies used are indicated in the bottom as ubiquitin (Ub), PhS_3_‐RNase, Ub‐K48 and Ub‐K63, respectively. K, lysine; FLAG, a protein tag. (b) Ubiquitination sites of PhS_3_‐RNase identified by LC‐MS/MS. Ub: the amino acid residue on its right within the peptide sequence of PhS_3_‐RNase is ubiquitinated. The b‐type and y‐type product ions are indicated. (c) The secondary structural features of PhS_3_‐RNase with the locations of the six ubiquitination sites. C1–C5, five conserved regions; Hva and Hvb, hypervariable region a and b. K, C and T: lysine, cysteine and threonine, respectively. (d) Spatial locations of the six ubiquitination sites on the three‐dimensional (3D) structure of PhS_3_‐RNase. The dark blue region indicates the Hv regions of PhS_3_‐RNase, the green region indicates the residues identified by LC‐MS/MS in unpollinated, self‐pollinated and cross‐pollinated pistils, and the cyan region indicates the residues identified specifically in cross‐pollinated pistils. I, II and III: three regions containing the ubiquitination sites shown in the predicted 3D structure of PhS_3_‐RNase.

Previous studies have revealed that S‐RNase is ubiquitinated in cross‐pollen tubes, but the linkage type or site of this ubiquitination remains unclear. To investigate these questions, we performed an *in vitro* ubiquitination assay and showed that both oligoubiquitinated and polyubiquitinated PhS_3_‐RNase were detected by anti‐ubiquitin, anti‐PhS_3_‐RNase and anti‐ubiquitin‐K48 antibodies compared with the *PhS_3_S_3L_* wild‐type control, indicating that nonself PhS_3L_SLF1 is capable of forming an SCF^SLF^ complex to ubiquitinate PhS_3_‐RNase mainly through K48‐linked polyubiquitin chains (Fig. 1a). To further detect the ubiquitination site of S‐RNase, we used LC‐MS/MS and identified six ubiquitinated residues at T102, K103, C118, T153, K154 and K217 positions of PhS_3_‐RNase from wild‐type pistils cross‐pollinated with the transgenic pollen containing the pollen‐specific *PhS_3L_SLF1* in *P*. *hybrida* (Figs 1b, S1). Furthermore, we found that the ubiquitinated C118, T153, K154 and K217 residues were exclusively detected in cross‐pollinated pistils, suggesting that they are specific for cross‐pollination, whereas the ubiquitinated T102 and K103 residues were detected in both unpollinated and self‐pollinated pistils (Fig. S2), suggesting that they are likely to occur before S‐RNase uptake into pollen tubes.

To determine the locations of these ubiquitinated amino acid residues in S‐RNases, we compared a total of 36 S‐RNases from Solanaceae and found that C118 was within the conserved (C) 3 region, T102 and K103 were adjacent to hypervariable (Hv) b, T153 and K154 were between C4 and C5 and K217 was found at the C‐terminal region, implying they are located in three largely conserved S‐RNase regions (Figs 1c, S3; Table S2). Next, we reasoned that the ubiquitination sites should be spatially close to E2. To examine this possibility, we determined the spatial localisation of six ubiquitinated residues on the predicted spatial structure of PhS_3_‐RNase and found that T102 and K103 residues were located near the Hvb region on an interface between S‐RNase and SLF and were termed region (R) I, T153, K154 and K217 were found in a region close to E2 and termed R II, whereas C118 was found inside the predicted spatial structure and was named R III (Fig. 1d). Taken together, our results demonstrated that S‐RNases are ubiquitinated mainly through K48 linkage at three largely conserved spatial regions among Solanaceae S‐RNases.

### Two ubiquitinated amino acids from R I are partially involved in PhS_3_‐RNase degradation during cross‐pollination

To examine how six ubiquitinated amino acids from three spatial regions mediate the S‐RNase ubiquitination, we first designed a mutant construct named MI containing T102A and K103R substitutions that was incapable of ubiquitination at R I of PhS_3_‐RNase and showed that its RNase activity increased with time, similar to the wild‐type (Fig. S4a), suggesting that MI possessed normal ribonuclease activity. To examine whether the substitutions affected the subcellular location of PhS_3_‐RNase, we performed fractionation experiments and found that MI was predominantly enriched in the S160 fraction derived from the pollen‐tube cytosol, similar to wild‐type PhS_3_‐RNase (S_3_R) (Fig. S4b). Furthermore, we performed pull‐down, SFLC and BiFC assays and found that MI was capable of interacting with nonself PhS_3L_SLF1 (Fig. S4c–e). Nevertheless, we also found that it displayed a weak interaction with self PhS_3_SLF1 (Fig. S5), similar to previous studies (Hua & Kao, 2006; Kubo *et al*., 2010). Consistent with these findings, we found that the predicted structure and electrostatic potentials of MI remain essentially unaltered (Fig. S6). Taken together, these results indicated that MI has an enzymatic activity and structure similar to wild‐type S_3_R.

To examine the *in vivo* function of MI, we transformed *S_3_R* and *MI* driven by the pistil‐specific *Chip* promotor into SI *PhS_3_S_3L_* plants, respectively and also transformed their *FLAG*‐tagged forms into *PhS_3_S_3L_*. For each construct, we identified at least 24 T_0_ transgenic lines by PCR analysis (Figs S7, S8) and found that *MI* was expressed normally in the transgenic lines (Figs S9, S10a,b). Furthermore, self‐pollination assays showed that each construct did not alter the SI phenotypes of the transgenic plants (Tables S3, S4). To examine their roles in cross‐pollination, we further identified several lines with similar transgene expression levels. Compared with *c*. 398 seed sets per capsule from *PhS_3_S_3L_* carrying the transgenic *S_3_R* (*S_3_S_3L_*/*S_3_R*‐60) pollinated with cross‐pollen of *PhS_V_S_V_*, *S_3_S_3L_*/*MI* had a reduced seed set of 298 with a reduction of 25% (Fig. S10c,d; Table S3). Consistent with this, we also found a substantial reduction of seed sets derived from cross‐pollination of the *FLAG*‐tagged transgenic line *S_3_S_3L_*/*MI*‐*FLAG*‐24 (292 per capsule) with a 30% reduction compared with 421 seeds per capsule from *S_3_S_3L_*/*S_3_R*‐*FLAG*‐34 (Fig. S10c,d; Table S4). Taken together, these results suggested that ubiquitinated R I was involved in cross‐pollination.

To verify this role, we assessed the degradation rates of recombinant SUMO‐His‐tagged S_3_R and MI proteins by cell‐free degradation assays using self‐ (*PhS_3_S_3_*) or cross‐ (*PhS_V_S_V_*) pollen‐tube extracts (PTE). The results showed that both SUMO‐His‐tagged S_3_R and MI remained essentially stable in *PhS_3_S_3_* PTE but SUMO‐His‐S_3_R degraded rapidly in the *PhS_V_S_V_* PTE without MG132 and only 7% remained after a 10‐min treatment (Fig. S10e,f). However, the degradation rates decreased slightly and *c*. 25% remained after a 10‐min treatment for SUMO‐His‐MI protein (Fig. S10f), showing that MI degradation was partially inhibited and, therefore, resulted in its accumulation in cross‐pollen tubes. Consistently, both S_3_R‐FLAG and MI‐FLAG remained largely stable in self‐ (*PhS_3_S_3_*) pollen tubes, but their amounts significantly decreased in cross‐ (*PhS_V_S_V_*) pollen tubes during semi *in vivo* pulse–chase experiments using style extracts of *S_3_S_3L_*/*MI*‐*FLAG*‐24 or *S_3_S_3L_*/*S_3_R*‐*FLAG*‐34 to mimic self‐pollination and cross‐pollination (Fig. S10g). Nevertheless, an additional *c*. 12% remained for MI‐FLAG in cross‐pollen tubes compared with S_3_R‐FLAG, suggesting that the cross‐pollen rejections by the transgenic pistils containing MI‐FLAG mainly resulted from its decreased degradation. In addition, degradation of these proteins by cross‐pollen was significantly delayed by MG132 treatment (Fig. S10e–g), indicating the degradation of MI by the UPS pathway similar to that found in wild‐type.

To confirm the role of the ubiquitinated R I in S‐RNase degradation, we further performed ubiquitination assays and found that by contrast with self‐ (*PhS_3_S_3_*) PTE, both polyubiquitinated His‐tagged S_3_R and MI proteins were detected by anti‐ubiquitin and anti‐His antibodies (Figs S11, S12) after incubation with SCF^PhS3LSLF1^ serving as E3, and indicating that they both could be specifically ubiquitinated by nonself SCF^PhS3LSLF1^. Nevertheless, the ubiquitinated products of MI reduced to *c*. 60% that of S_3_R (Fig. S11), suggesting that the ubiquitinated residues located in R I were partially responsible for the ubiquitination of PhS_3_‐RNase by cross‐pollen. Taken together, these results revealed that two ubiquitinated amino acids from R I were partially involved in PhS_3_‐RNase degradation during cross‐pollination.

### The R II from PhS_3_‐RNase serves as a major region for its ubiquitination and degradation in cross‐pollen tubes

**Fig. 2:**
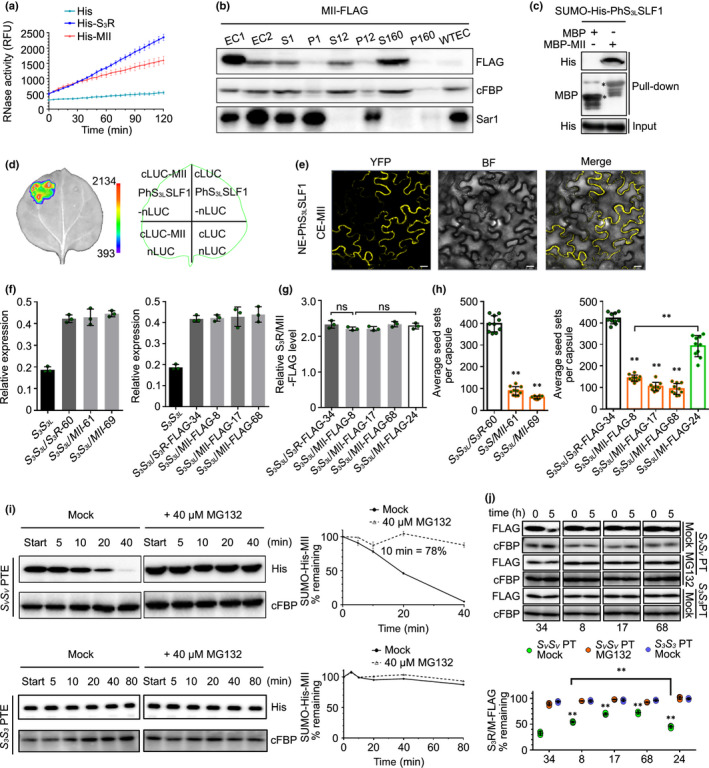
*Petunia hybrida* S_3_‐RNase with mutated region (R) II significantly inhibits cross seed sets. (a) RNase activity detection of His‐S_3_R and mutant (M) II expressed by *pCold*‐*TF* vectors. The relative fluorescence unit (RFU) indicating RNase activity during a time‐course experiment is shown as mean ± SD (*n* = 3). II: the ubiquitinated region II of PhS_3_‐RNase. (b) Immunoblot detection of FLAG‐tagged MII in subcellular fractions of *in vitro* germinated pollen tubes. EC1, EC2 and WTEC indicate entire cell homogenates of the pistils from the transgenic plants containing *MII*‐*FLAG*, the pollen tubes of *PhS_V_S_V_* treated with EC1 and the pistils from wild‐type *PhS_3_S_3L_*. WTEC was a negative control. S1 and P1, S12 and P12, S160 and P160 indicate supernatant and pellet fractions obtained by centrifugation of EC2 at 1000 ***g***, 12 000 ***g*** and 160 000 ***g***, respectively. cFBP and Sar1 are respective marker antibodies of cytosol and endoplasmic reticulum (ER). (c) Physical interactions between PhS_3L_SLF1 and MII detected by pull‐down assay. Input and pull‐down: bait protein SUMO‐His‐PhS_3L_SLF1 and prey proteins detected by immunoblots, respectively. MBP and SUMO‐His are protein tags. Asterisks indicate bands of target proteins. (d) Split firefly luciferase complementation (SFLC) assay. The numbers on the left side of the colour signal bars represent the values of the fluorescent signal. The injection positions of each component on tobacco leaves are indicated in the contour diagram of leaf margin. nLUC and cLUC indicate transiently expressed N‐terminal and C‐terminal regions of luciferase. (e) Bimolecular fluorescence complementation (BiFC) assay. NE and CE: transiently expressed N‐terminal and C‐terminal regions of YFP by *pSPYNE* and *pSPYCE* vectors. YFP, BF and Merge represent the YFP fluorescence, bright field and their merged field, respectively. Bars, 20 μm. (f) Transcripts of the transgene and native *PhS_3_*‐*RNase* detected by qRT‐PCR. The T_0_ transgenic lines are indicated below the horizontal axes. *S_3_S_3L_* is a wild‐type. Data are shown as mean ± SD (*n* = 3). (g) Quantitative analyses of S_3_R‐ and MII‐FLAG proteins. The T_0_ transgenic lines are indicated below the horizontal axes. Data are shown as mean ± SD (*n* = 3). Student’s *t*‐test was used to generate the *P‐*values. ns (not significant), *P* > 0.05. (h) Statistical analyses of seed sets per capsule from T_0_ transgenic plants pollinated with cross‐pollen of *PhS_V_S_V_*. Data are shown as mean ± SD (*n* ≥ 9). Student’s *t*‐test was used to generate the *P‐*values. **, *P* < 0.01. (i) Cell‐free degradation of recombinant SUMO‐His‐MII by pollen‐tube extracts (PTE) of *PhS_V_S_V_* or *PhS_3_S_3_*. Left, immunoblots of the reaction products incubated with or without MG132 (Mock). Start, time point zero in each degradation assay. cFBP antibody was used to detect nondegraded loading control. Right, quantitative analyses of the degradation rates. Data are shown as mean ± SD (*n* = 3). The remaining amount at 10 min is indicated. (j) Time‐course analyses of PhS_3_R‐FLAG and MII‐FLAG levels in the cross‐pollen tubes (PTs) (*PhS_V_S_V_*) or self‐PTs (*PhS_3_S_3_*) incubated with or without MG132 (Mock). *PhS_V_S_V_* and *PhS_3_S_3_* PTs were challenged with style extracts of *PhS_3_S_3L_*/*PhS_3_R*‐*FLAG* or *PhS_3_S_3L_*/*MII*‐*FLAG* for 5 h to mimic cross‐pollination and self‐pollination, respectively. Top, immunoblots of PhS_3_R‐FLAG or MII‐FLAG in the PT using FLAG antibody. cFBP was detected as a loading control. The numbers at the bottom indicate the transgenic line numbers corresponding to those in (h). Bottom, quantitative analyses of the immunoblots. Data are shown as mean ± SD (*n* = 3). Student’s *t*‐test was used to generate the *P‐*values. **, *P* < 0.01.

To examine the function of three ubiquitinated amino acids from R II, we followed a similar strategy to that used for R I by creating MII with T153A, K154R and K217R substitutions of PhS_3_‐RNase. We found that its RNase activity increased with time, similarly to that found for wild‐type (Fig. 2a), suggesting that it possessed normal ribonuclease activity. Second, it showed that MII was also predominantly located in the pollen‐tube cytosol (Fig. 2b), capable of interacting with nonself PhS_3L_SLF1 (Fig. 2c–e), also with a weak interaction with self PhS_3_SLF1 (Fig. S5) and had unaltered predicted structure and electrostatic potentials (Fig. S6). Furthermore, *MII* and its *FLAG*‐tagged transgenes were expressed normally in SI *PhS_3_S_3L_* plants, and the transgenic lines also maintained an SI phenotype (Figs 2f,g, S7–S9; Tables S3, S4). Compared with *MI* transgenic lines, a significant difference observed for *S_3_S_3L_*/*MII* was the seed sets derived from pollination with cross‐pollen of *PhS_V_S_V_* (*c*. 75 seed sets per capsule), with a significant reduction of 81% compared with *S_3_S_3L_*/*S_3_R*‐60 (398 seed sets per capsule) (Figs 2h, S13a; Table S3). Consistent with this result, compared with 421 seeds per capsule from *S_3_S_3L_*/*S_3_R*‐*FLAG*‐34, *c*. 113 seeds were set for the *FLAG*‐tagged transgenic lines with a significant 73% reduction (Figs 2h, S13b; Table S4). We further found that the *MII*‐*FLAG* transgene led to the production of much fewer seed sets per capsule than *MI*‐*FLAG* whereas their protein levels remained similar (Figs 2g,h, S13b), indicating that the ubiquitinated R II plays a major role in cross‐pollination. Furthermore, cell‐free degradation assays and pulse–chase experiments mimicking cross‐pollination showed that the degradation of MII protein was mainly through the UPS pathway and was severely inhibited, with a significantly decreased difference between its remaining amounts in self‐pollen and cross‐pollen tubes compared with MI (Figs 2i,j, S10f). Consistently, *in vitro* ubiquitination assays showed that the ubiquitination amount of MII with SCF^PhS3LSLF1^ serving as E3 significantly reduced to 40% of S_3_R, with a reduction of 20% compared with MI (Fig. S11). In addition, both the attenuated degradation and ubiquitination of MII specifically occurred in cross‐pollen tubes (Figs 2i,j, S11, S12). Taken together, these results suggested that R II of PhS_3_‐RNase acts as a major ubiquitination region for its degradation resulting in cross‐pollination.

### K154 and K217 from R II act as two major ubiquitination residues for PhS_3_‐RNase degradation in cross‐pollen tubes

**Fig. 3:**
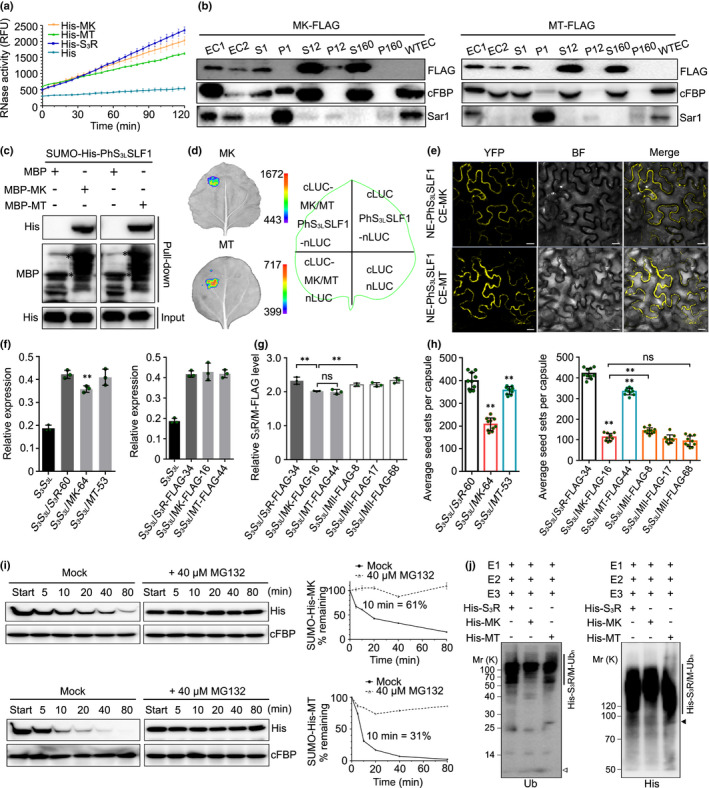
K154 and K217 of the region (R) II of *Petunia hybrida* S_3_‐RNase serve as two major ubiquitination sites for its degradation in cross‐pollen tubes. (a) RNase activity detection of His‐mutant (M) K and MT expressed by *pCold*‐*TF* vectors. RFU: relative fluorescence unit. K and T: lysine and threonine within six ubiquitinated residues of PhS_3_‐RNase. Data represent mean ± SD (*n* = 3). (b) Immunoblot detection of FLAG‐tagged MK and MT in subcellular fractions of *in vitro* germinated pollen tubes. EC1, EC2 and WTEC: entire cell homogenates of the pistils from the transgenic plants containing *MK*‐*FLAG* or *MT*‐*FLAG*, the pollen tubes of *PhS_V_S_V_* treated with EC1 and the pistils from wild‐type *PhS_3_S_3L_*. S1, P1, S12, P12, S160 and P160: supernatant and pellet fractions obtained by centrifugation of EC2 at 1000 ***g***, 12 000 ***g*** and 160 000 ***g***, respectively. cFBP and Sar1: respective marker antibodies of cytosol and endoplasmic reticulum (ER). (c) Physical interactions between PhS_3L_SLF1 and MK and MT detected by pull‐down assays. MBP and SUMO‐His: protein tags. (d) SFLC assays. nLUC and cLUC: transiently expressed N‐terminal and C‐terminal regions of luciferase. (e) BiFC assays. NE and CE: transiently expressed N‐terminal and C‐terminal regions of YFP. YFP, BF and Merge: the YFP fluorescence, bright field and their merged field, respectively. Bars, 20 μm. (f) Transcripts of transgene and native *PhS_3_*‐*RNase* detected by qRT‐PCR. Data are shown as mean ± SD (*n* = 3). Student’s *t*‐test: **, *P* < 0.01. (g) Quantitative analyses of S_3_R‐FLAG, MK‐FLAG and MT‐FLAG proteins. Data are shown as mean ± SD (*n* = 3). Student’s *t*‐test: ns, not significant; *P* > 0.05; **, *P* < 0.01. (h) Statistical analyses of seed sets per capsule from T_0_ transgenic plants pollinated with cross‐pollen of *PhS_V_S_V_*. Data are shown as mean ± SD (*n* = 10). Student’s *t*‐test: ns (not significant), *P* > 0.05; **, *P* < 0.01. (i) Immunoblots of recombinant SUMO‐His‐MK and ‐MT in the cell‐free degradation products incubated by *PhS_V_S_V_* pollen‐tube extracts (PTE) with or without MG132 (Mock). Data are shown as mean ± SD (*n* = 3). (j) Immunoblot detection of *in vitro* ubiquitination products of His‐S_3_R, ‐MK and ‐MT by SCF^PhS3LSLF1‐FLAG^ (E3) using anti‐ubiquitin (Ub) and anti‐His antibodies. The vertical lines illustrate the ubiquitinated substrates. Open and closed arrowheads indicate ubiquitin and unubiquitinated substrate monomers, respectively. Annotations of this figure are identical to those of Fig. 2.

To explore the function of three lysines (K103, K154 and K217) and two threonines (T102 and T153) residues of PhS_3_‐RNase in its degradation, we designed two mutant constructs termed MK (K103R, K154R and K217R) and MT (T102A and T153A), which showed similar enzymatic activities, subcellular localisations, SLF interactions, predicted structures and electrostatic potentials to wild‐type S_3_R as well as normal pistil expressions and SI phenotypes similar to SI *PhS_3_S_3L_* plants (Figs 3a–g, S5–S9; Tables S3, S4). However, *MK* and *MT* transgenic lines showed differential seed sets of 207 and 356 per capsule after pollination with cross‐pollen of *PhS_V_S_V_*, with a significant reduction of 48% and 15%, respectively, compared with *S_3_S_3L_*/*S_3_R*‐60 (398 seeds per capsule) (Figs 3h, S14a; Table S3). This was consistent with the *S_3_S_3L_*/*MK*‐*FLAG*‐16 set with 113 seeds per capsule with a significant reduction of 73% and 66% compared with *S_3_S_3L_*/*S_3_R*‐*FLAG‐*34 (421 seeds per capsule) and *S_3_S_3L_*/*MT*‐*FLAG‐*44 (334 seeds per capsule), respectively (Figs 3h, S14b; Table S4). In addition, we showed that, compared with the *MT* transgene, *MK* resulted in a greater seed set reduction similar to that found for *MII* when the transgene expression levels were similar (Fig. 3g,h), suggesting that the identified lysine amino acids, especially K154 and K217, played a major role in the ubiquitination and degradation of PhS_3_‐RNase. Furthermore, cell‐free degradation and pulse–chase assays showed that MK degradation by the 26S proteasome in cross‐ (*PhS_V_S_V_*) pollen tubes had been significantly delayed compared with MT in the absence of MG132 (Figs 3i,S14c,d). Ubiquitination assays also indicated that lysine residues rather than threonine residues act as the major sites for PhS_3_‐RNase ubiquitination by nonself SCF^PhS3LSLF1^ (Figs 3j, S12). Taken together, our results suggested that K154 and K217 from R II functioned as two major ubiquitination residues of PhS_3_‐RNase for cross‐pollination.

### R III functions as the second major ubiquitination region for PhS_3_‐RNase degradation allowing cross‐pollination

To investigate the function of the ubiquitination site C118 from the internal R III, we designed MIII (C118A) and found that it also maintained ribonuclease activity, subcellular localisation and predicted structure similar to the wild‐type S_3_R (Figs S5, S6, S15). We further transformed *MIII* and its *FLAG*‐tagged form into SI *PhS_3_S_3L_* plants and detected significantly reduced numbers of seed sets of *c*. 160 and 261 per capsule from *S_3_S_3L_*/*MIII*‐84 and *S_3_S_3L_*/*MIII*‐*FLAG*‐18 after pollination with cross‐pollen of *PhS_V_S_V_*, with a respective reduction of 59% and 38% compared with *S_3_S_3L_*/*S_3_R*‐60 and *S_3_S_3L_*/*S_3_R*‐*FLAG*‐34 (Figs S7–S9, S16a–d; Tables S3, S4). Furthermore, the average seed set number per capsule was much fewer than that for *S_3_S_3L_*/*MI*‐*FLAG* when they showed similar transgene expression levels (Fig. S16b,d), supporting a role for R III in the degradation of PhS_3_‐RNase. In addition, we detected a marked accumulation of MIII in cross‐pollen tubes compared with S_3_R and MI in the absence of MG132 (Figs S10e–g, S16e,f) and a significantly decreased level of ubiquitination by nonself SCF^PhS3LSLF1^ compared with MI (Figs S11, S12). Taken together, these results suggested that R III acts as a second major ubiquitination region for the degradation of PhS_3_‐RNase, therefore leading to cross‐pollination.

### R I, R II and R III of PhS_3_‐RNase function additively in its degradation during cross‐pollination

**Fig. 4:**
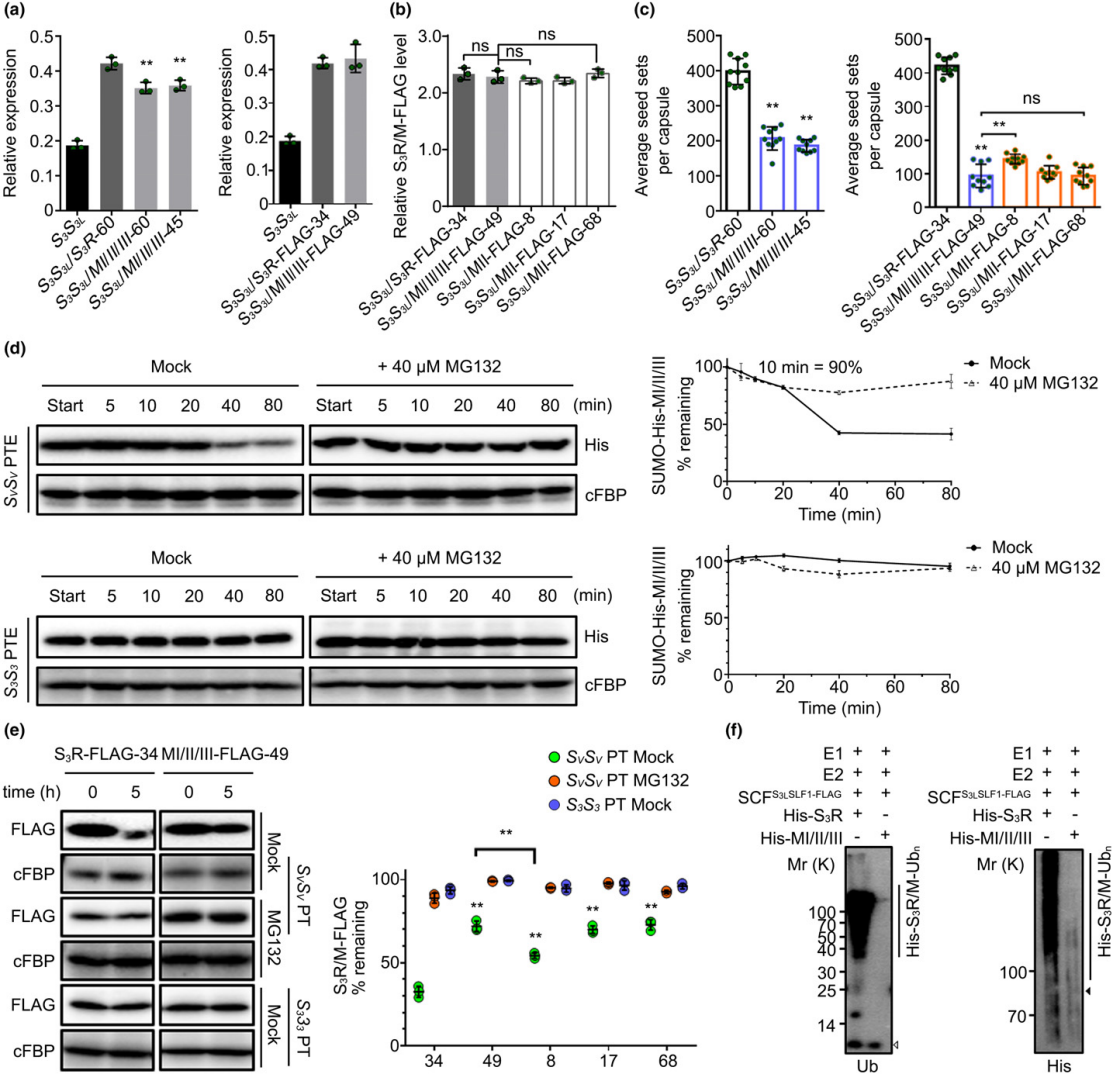
*Petunia hybrida* S_3_‐RNase with mutated region (R) I, II and III regions significantly reduces cross seed sets. (a) Transcripts of the transgene (*PhS_3_*‐*RNase* or *mutant* (*M*) *I*/*II*/*III*) and native *PhS_3_*‐*RNase* detected by qRT‐PCR. Data are shown as mean ± SD (*n* = 3). Student’s *t*‐test: **, *P* < 0.01. I, II and III, three ubiquitination regions of PhS_3_‐RNase. FLAG, a protein tag. (b) Quantitative analyses of S_3_R‐FLAG and MI/II/III‐FLAG proteins. Data are shown as mean ± SD (*n* = 3). Student’s *t*‐test: ns (not significant), *P* > 0.05. (c) Statistical analyses of seed sets per capsule from T_0_ transgenic lines containing *MI*/*II*/*III* pollinated with cross‐pollen of *PhS_V_S_V_*. Data are shown as mean ± SD (*n* = 10). Student’s *t*‐test: ns (not significant), *P* > 0.05; **, *P* < 0.01. (d) Immunoblots of recombinant SUMO‐His‐MI/II/III in the cell‐free degradation products incubated in the pollen‐tube extracts (PTE) of *PhS_V_S_V_* or *PhS_3_S_3_* with or without MG132 (Mock). Data represent mean ± SD (*n* = 3). (e) Time‐course analyses of MI/II/III‐FLAG amounts in the cross‐ (*PhS_V_S_V_*) or self‐ (*PhS_3_S_3_*) pollen tubes (PT) incubated with or without MG132. Data are shown as mean ± SD (*n* = 3). Student’s *t*‐test: **, *P* < 0.01. (f) Immunoblot detections of *in vitro* ubiquitinated recombinant His‐tagged S_3_R and MI/II/III. Annotations of this figure are identical to those of Figs 2 and 3.

To examine the function of the three ubiquitination regions together, we made MI/II/III (T102A, K103R, T153A, K154R, K217R and C118A). Similar to wild‐type PhS_3_‐RNase, the mutant form exhibited normal physicochemical properties (Figs S5, S6, S17) but resulted in 197 and 93 cross seeds per capsule derived from *S_3_S_3L_*/*MI*/*II*/*III*‐45 and *S_3_S_3L_*/*MI*/*II*/*III*‐*FLAG*‐49 with *PhS_V_S_V_* pollen, respectively, a significant reduction of 50% and 77%, and similar to the lines containing mutated R II (Figs 4a–c, S7–S9, S18; Tables S3, S4). Furthermore, the degradation of MI/II/III in cross‐pollen tubes was strongly inhibited in the absence of MG132 (Fig. 4d,e), indicating a significantly reduced ubiquitination by nonself SCF^PhS3LSLF1^ in cross‐pollen (Figs 4f, S12). Taken together, these results suggested that the degradation of PhS_3_‐RNase was largely dependent on an additive role of its three ubiquitination regions during cross‐pollination.

## Discussion

**Fig. 5:**
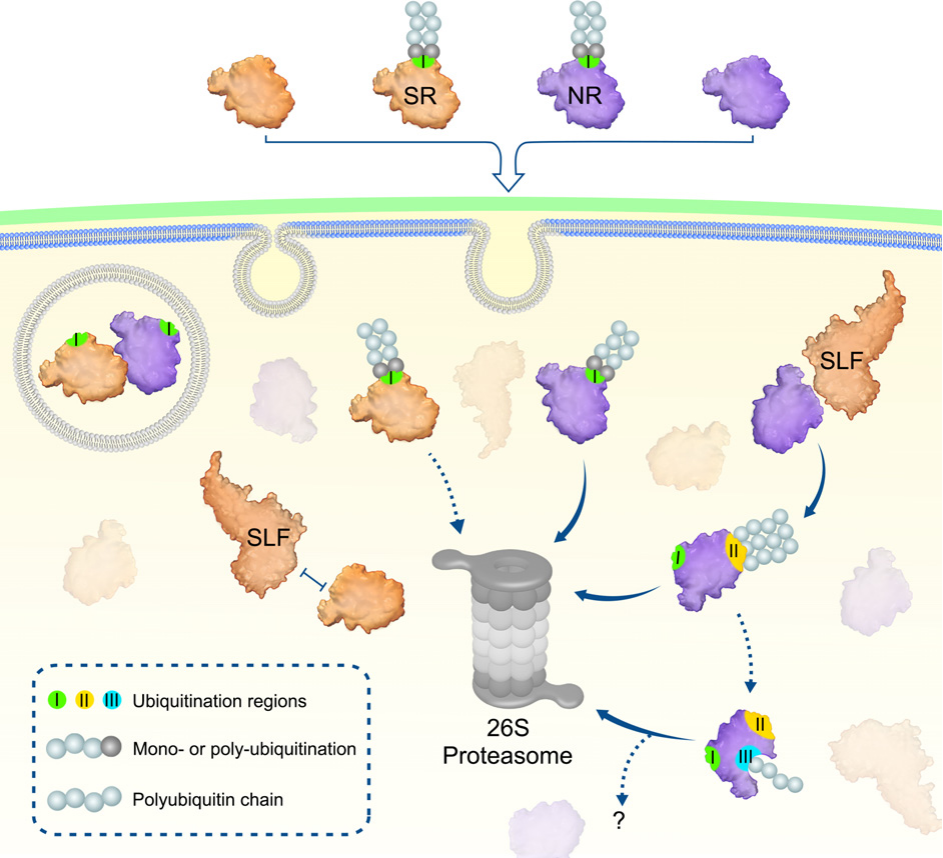
Proposed model for a stepwise ubiquitination and degradation mechanism of *Petunia hybrida* S‐RNases. Self and nonself S‐RNases (SR and NR) and their region (R) I ubiquitinated forms can enter the cytoplasm through the pollen‐tube membrane. The R I ubiquitinated S‐RNases could be degraded by the 26S proteasome, whereas their unubiquitinated forms are identified by SLFs. SR and its cognate SLF repel each other and results in an inability of SCF^SLF^ to ubiquitinate SR. By contrast, NR is attracted by nonself SLF for R II ubiquitination by SCF^SLF^ and its subsequent degradation by the 26S proteasome. Subsequently, internal R III could be further exposed for ubiquitination, leading to degradation of NR by the 26S proteasome or other unknown pathways. In addition, S‐RNase compartmentalisation could occur in the vacuole and contribute to its sequestration. I, II, and III: three ubiquitination regions in S‐RNases.

Previous studies have shown that nonself S‐RNases are collaboratively recognised by multiple nonself SLFs leading to the formation of canonical SCF^SLF^ complexes for their ubiquitination and subsequent degradation by the 26S proteasome during cross‐pollination, but the ubiquitination linkage type and site remain unclear. In this study, we found that nonself S‐RNase is mainly polyubiquitinated through K48 linkages by SCF^SLF^ at three spatial regions (R I, R II and R III) in *P*. *hybrida*. Among them, R I ubiquitination appears to occur before S‐RNase uptake into pollen tubes with a minor role, if any, in cross‐pollen tubes, whereas R II and R III act as two major ubiquitination regions for S‐RNase degradation. Consistently, Hua & Kao (2008) identified six lysine residues mainly responsible for the ubiquitination and degradation of S_3_‐RNase in *P*. *inflata*, which are located in the same region of R II identified in our study, reinforcing the major role of R II in *Petunia* S_3_‐RNase degradation for cross‐pollination. Based on our results, we propose a stepwise UPS model for S‐RNases cytotoxicity restriction allowing cross‐pollination in *P*. *hybrida* (Fig. 5). In this model, both self and nonself S‐RNases with a small fraction of R I ubiquitinated forms are likely to be mediated by an unknown E3 ligase and are taken up into the cytosols of either self‐pollen or cross‐pollen tubes. First, the R I ubiquitinated forms would make them unable to be recognised by SLFs but degraded by the 26S proteasome. Second, other S‐RNases could be recognised by SLFs on the basis of ‘like charges repel and unlike charges attract’, and the like electrostatic potentials together with other unknown forces between self S‐RNase and its cognate SLF would result in the formation of nonfunctional SCF^SLF^ complexes as demonstrated previously (Li *et al*., 2017). By contrast, nonself S‐RNase would be attracted by unlike electrostatic potentials and other unknown factors and polyubiquitinated by functional SCF^SLF^ complexes (Li *et al*., 2017; Sun & Kao, 2018) at R II, leading to its degradation by the 26S proteasome. Thirdly, the internal R III of nonself S‐RNase could be exposed by a conformational change for its further ubiquitination by SLFs and degradation resulting in cross‐pollination. Our studies have revealed that the ubiquitination and degradation of nonself S‐RNases depend on at least three regions with distinct ubiquitination sites, including lysine, threonine and cysteine, reinforcing the notion that the restriction of S‐RNase cytotoxicity occurs mainly by the ubiquitination‐mediated degradation mechanism in *P*. *hybrida* (Liu *et al*., 2014).

Nevertheless, the underlying mechanisms of R I and R III ubiquitination remain to be further elucidated. Notably, newly synthesised secretory proteins are constantly scrutinised and destructed by protein quality control systems such as ER‐associated degradation (ERAD) or autophagy to maintain proteostasis once they are misfolded or aggregated (Anelli & Sitia, 2008). The ubiquitination of R I in the unpollinated pistils suggested that it might result from the polyubiquitination of misfolded PhS_3_‐RNases. Ubiquitination of closely spaced residues is predicted to be important for polyubiquitin chain assembly (Wang *et al*., 2012). Here, we found that the identified threonine ubiquitination residues paired with lysine also contributed to the ubiquitination and degradation of nonself S‐RNase for cross‐pollination, suggesting that they may play a role in building polyubiquitin chains long enough for proteasome recognition (Thrower *et al*., 2000). In addition, ubiquitin often serves as a critical signal governing the membrane traffic system. Monoubiquitination is sufficient to initiate the internalisation of plasma membrane proteins, and K63‐linked polyubiquitination is frequently involved in their subsequent sorting and trafficking (Hicke & Dunn, 2003; Clague & Urbé, 2010; Paez Valencia *et al*., 2016). It is therefore possible that R I could also be monoubiquitinated leading to S‐RNase entry into pollen tubes by endocytosis, consistent with the results showing that a small fraction of S‐RNases is sequestered in microsome fractions (Liu *et al*., 2014). As for the internal R III, its ubiquitination is likely to occur only after being exposed. Therefore, the ubiquitination of R II might lead to the conformational change of nonself S‐RNase and exposure of R III, and the subsequent ubiquitination of R III would further block the enzymatic activity of the S‐RNase (Sagar *et al*., 2007). Previous simulations have demonstrated that the conserved complementary electrostatic patterns and hydrophobic patches of Rpn10, a recognition subunit of proteasome, and K48‐linked tetraubiquitin of the substrates, are critical for their interaction (Zhang *et al*., 2016). Similarly, the ubiquitination of R III might further enhance the electrostatic potentials and hydrophobicity to strengthen the recognition of ubiquitinated S‐RNase by the proteasome as well as its degradation.

It remains unclear why the additive action of the three regions did not completely restrict nonself S‐RNase cytotoxicity in cross‐pollen tubes. We suggest that there might be additional mechanism (s) occurring either after the R III‐mediated ubiquitination of nonself S‐RNase or during S‐RNase uptake into pollen tubes by endocytosis resulting in its compartmentalisation (Goldraij *et al*., 2006). In *Nicotiana*, S‐RNases appeared to be sequestered early in the vacuole but released out late to the cytosol in self‐pollen tubes (Goldraij *et al*., 2006). In *P*. *hybrida*, a small amount of S‐RNases was detected in the microvesicles of pollen tubes (Liu *et al*., 2014). Furthermore, it has been shown that S‐RNases were compartmentalised in the cross‐pollen tubes even 36 h after cross‐pollination in *Nicotiana* (Goldraij *et al*., 2006), longer than 24 h, the longest post‐pollination time used for identification of R I/R II/R III ubiquitination in *P*. *hybrida*. Together, these findings suggested that S‐RNase compartmentalisation and its stepwise ubiquitination and degradation might function synergistically from the beginning to the late phases of S‐RNase action in the cross‐pollen tubes. In this scenario, it is possible that the mutant S‐RNase evades ubiquitination and degradation and that would partially reverse the downstream response derived from nonself‐recognition between SLFs and S‐RNases to that of their self‐recognition. This would further lead to S‐RNase release from the vacuole to varying degrees, similar to the late stage of self‐pollen rejection proposed in *Nicotiana* (Goldraij *et al*., 2006). In this view, the increased S‐RNase cytotoxicity could result from the total effects of S‐RNases, both originally present in and later released from the vacuole into the cross pollen‐tube cytosol. Nevertheless, removal of the additive ubiquitination of R I/R II/R III could not lead to sufficient cytosolic S‐RNases, therefore allowing the cross‐pollen to escape and to seed sets. Further investigation into the S‐RNase uptake mechanism and the relationship between the stepwise ubiquitination and degradation of S‐RNases and their compartmentalisation would provide the answer to these possibilities.

T2 RNases are widespread in every organism except Archaea and are involved in a variety of biological processes, including phosphate starvation, viral infection, self‐fertilisation, tumour growth control and cell death (Löffler *et al*., 1992; Bariola *et al*., 1994; Meyers *et al*., 1999; Thompson & Parker, 2009; Ramanauskas & Igić, 2017). However, our understanding of their function remains largely incomplete, especially when their roles appear to be independent of their enzymatic activity. In *Saccharomyces cerevisiae*, T2 RNase Rny1 can be released from the vacuole to cleave tRNA and rRNA under superoxygen stress (Thompson & Parker, 2009). Rny1 is indispensable for cell viability, but overexpressed Rny1 can act as a cytotoxin during oxidative stress (MacIntosh *et al*., 2001; Thompson & Parker, 2009). Moreover, its inactivation strikingly has no effects on cell viability (MacIntosh *et al*., 2001), but the underlying mechanism remains elusive. In human, RNASET2 is not only implicated to regulate neurodevelopment downstream of the immune response, but also serve as a tumour suppresser (Henneke *et al*., 2009), whereas how it contributes to this process in a cleavage‐independent manner is poorly defined. In addition, the catalytic‐independent function of T2 RNase has also been confirmed for ACTIBIND from *Aspergillus niger* that can bind to and destroy the normal actin networks, and is supposed to be conserved in other T2 RNase family members including S‐RNases (Roiz *et al*., 2006). Therefore, T2 RNase may act as a molecular signal mediating multiple biological settings, revealing that diverse T2 RNase roles could be derived through neofunctionalisation in these lineages.

In S‐RNase‐based SI, Yang *et al*. (2018) reported an S‐RNase‐mediated actin disruption in apple (*Malus* × *domestica*). The disrupted cytoskeleton dynamic served as a major cause of PCD (Thomas *et al*., 2006), which is also proposed to occur in self‐pollen tubes of *Pyrus bretschneideri* (Chen *et al*., 2018). Moreover, self S‐RNase can disrupt Ca^2+^ gradients at the pollen‐tube apex by inhibiting phospholipase C (PLC) (Qu *et al*., 2017). In addition, heat‐inactivated S‐RNase surprisingly exerts a more severe inhibition of pollen tubes (Gray *et al*., 1991). These studies suggested that S‐RNase could function in a signalling pathway independent of its enzymatic activity. Our results indicated that self S‐RNase could be partially ubiquitinated extracellularly and destroyed during its uptake, but we cannot rule out the possibility that its ubiquitination could act as an initial signal for the SI response.

In addition to ubiquitination, a recent study in *Solanum chacoense* showed that the number of carbohydrate chains of S‐RNase may influence its threshold for pollen rejection (Liu *et al*., 2008). Torres‐Rodriguez *et al*. (2020) found that the ribonuclease activity of S_C10_‐RNase could be significantly enhanced if its conserved Cys155–Cys185 disulphide bond was reduced by *Nicotiana alata* thioredoxin type h (Natrxh) in the pollen‐tube cytosols. Furthermore, the disulphide bond of S_C10_‐RNase is highly conserved among S‐RNases (Anderson *et al*., 1989), and it is likely that S‐RNase reduction occurs after incompatible recognition between SLFs and S‐RNases, leading to RNA degradation necessary for self‐pollen rejection. In this case, the mutant S‐RNases incapable of ubiquitination and degradation in the cytosols of cross‐pollen tubes could induce downstream responses, including S‐RNase reduction, resulting in its increased ribonuclease activity as well as cytotoxicity for cross‐pollen inhibition. Moreover, as phosphorylation serves as a critical modification modulating multiple cellular events, it may also be involved in S‐RNase activity regulation and the downstream signalling transduction in Solanaceae‐type SI. In addition, previous studies have shown that other factors, except electrostatic potentials, should exist that contribute to the recognition between SLF and S‐RNase (Li *et al*., 2017). Therefore, future studies on the structure of SLF bound to S‐RNase, and other post‐translational modifications such as glycosylation, reduction and phosphorylation of S‐RNase and their relationships with its ubiquitination should shed light on how S‐RNase functions and stimulates downstream signalling networks in the pollen tubes.

In conclusion, our results have revealed a novel stepwise UPS mechanism for S‐RNase cytotoxicity restriction resulting in cross‐pollination in *P*. *hybrida*. Our findings also indicated a possible mechanism for dynamic regulation of secreted cytotoxin activities including other T2 ribonuclease members. Further validation of this mechanism using biochemical and cytological approaches is expected to provide additional insights into UPS and Solanaceae‐type SI.

## Author contributions

YX conceived and designed the project. HZ and YS performed the experiments. JL and YZ conducted functional analyses of *PhS_3L_SLF1*. HH assisted transgenic plant construction. QL and YZ provided technical support. HZ and YX analysed data and wrote the manuscript. All authors commented on the article. HZ and YS contributed equally to this work.

## Supporting information

**Fig. S1** Identification of six ubiquitinated residues of *Petunia hybrida* S_3_‐RNase by LC‐MS/MS analysis of cross‐pollinated pistils.
**Fig. S2** Two ubiquitinated residues of *Petunia hybrida* S_3_‐RNase identified by LC‐MS/MS analysis of self‐pollinated and unpollinated pistils.
**Fig. S3** Locations of six ubiquitinated residues of *Petunia hybrida* S_3_‐RNase in Solanaceous S‐RNases.
**Fig. S4***Petunia hybrida* S_3_‐RNase with the mutated region (R) I displays largely unaltered biochemical and physical properties.
**Fig. S5** Physical interactions between *Petunia hybrida* S_3_R/Mutant (M) and PhS_3_SLF1.
**Fig. S6** Predicted three‐dimensional (3D) structures and surface electrostatic potentials of *Petunia hybrida* S_3_‐RNases with mutated ubiquitinated residues.
**Fig. S7***Petunia hybridaS_3_R* and *S_3_R* (*Mutant*) (*M*) transgenes identification by PCR analysis.
**Fig. S8** Identification of *FLAG*‐tagged *Petunia hybrida* *S_3_R* and *S_3_R* (*Mutant*) (*M*) transgenes by PCR analysis.
**Fig. S9** Detection of *FLAG*‐tagged *Petunia hybrida* *S_3_R* and *S_3_R* (*Mutant*) (*M*) transgenes expression by immunoblots.
**Fig. S10***Petuniahybrida* S_3_‐RNase with the mutated region (R) I slightly reduces cross seed sets.
**Fig. S11** Decreased ubiquitination amount of mutant (M) I, II and III mediated by SCF^S3LSLF1^ of *Petunia hybrida*.
**Fig. S12** Ubiquitination of *Petunia hybrida* S_3_R and its mutant forms by self‐ (*PhS_3_S_3_*) pollen‐tube extracts (PTE).
**Fig. S13** Reduced seed set per capsule from T_0_ transgenic lines with mutated region (R) II of *Petunia hybrida* S_3_‐RNase.
**Fig. S14** Reduced seed set per capsule from T_0_ transgenic lines with mutated lysine or threonine within the six ubiquitinated residues of *Petunia hybrida* S_3_‐RNase.
**Fig. S15** Mutant **(**M) III largely maintains the biochemical and physical properties of *Petunia hybrida* S_3_‐RNase.
**Fig. S16***Petuniahybrida* S_3_‐RNase with mutated region (R) III markedly reduces cross seed sets.
**Fig. S17** Largely unaltered physicochemical properties of *Petunia hybrida* S_3_‐RNase with mutated region (R) I, II and III.
**Fig. S18** Reduced seed set per capsule from T_0_ transgenic lines with mutated region (R) I/II/III of *Petunia hybrida* S_3_‐RNase.
**Table S1** List of primer sequences.
**Table S2** Names and accession numbers of S‐RNases used in this study.
**Table S3** Seed sets of *S_3_S_3L_*/*S_3_R* and *S_3_S_3L_*/*S_3_R* (*Mutant*) (*M*) T_0_ transgenic plants of *Petunia hybrida*.
**Table S4** Seed sets of *S_3_S_3L_*/*S_3_R*‐*FLAG* and *S_3_S_3L_*/*S_3_R* (*Mutant*) (*M*)‐*FLAG* T_0_ transgenic plants of *Petunia hybrida*.
Please note: Wiley Blackwell are not responsible for the content or functionality of any Supporting Information supplied by the authors. Any queries (other than missing material) should be directed to the *New Phytologist* Central Office.

## Data Availability

The data supporting the findings of this work are available from the corresponding author upon request.
