# Supplementary material for: Primary restriction of S‐RNase cytotoxicity by a stepwise ubiquitination and degradation pathway in Petunia hybrida
Source: New Phytol. 2021 May 30;231(3):1249–64. doi: 10.1111/nph.17438 (PMC8361771; doi:10.1111/nph.17438)
Supplement: Supplementary file 1 — Fig. S1 Identification of six ubiquitinated residues of Petunia hybrida S3‐RNase by LC‐MS/MS analysis of cross‐pollinated pistils. Fig. S2 Two ubiquitinated residues of Petunia hybrida S3‐RNase identified by LC‐MS/MS analysis of self‐pollinated and unpollinated pistils. Fig. S3 Locations of six ubiquitinated residues of Petunia hybrida S3‐RNase in Solanaceous S‐RNases. Fig. S4Petunia hybrida S3‐RNase with the mutated region (R) I displays largely unaltered biochemical and physical properties. Fig. S5 Physical interactions between Petunia hybrida S3R/Mutant (M) and PhS3SLF1. Fig. S6 Predicted three‐dimensional (3D) structures and surface electrostatic potentials of Petunia hybrida S3‐RNases with mutated ubiquitinated residues. Fig. S7Petunia hybridaS3R and S3R (Mutant) (M) transgenes identification by PCR analysis. Fig. S8 Identification of FLAG‐tagged Petunia hybrida S3R and S3R (Mutant) (M) transgenes by PCR analysis. Fig. S9 Detection of FLAG‐tagged Petunia hybrida S3R and S3R (Mutant) (M) transgenes expression by immunoblots. Fig. S10Petuniahybrida S3‐RNase with the mutated region (R) I slightly reduces cross seed sets. Fig. S11 Decreased ubiquitination amount of mutant (M) I, II and III mediated by SCFS3LSLF1 of Petunia hybrida. Fig. S12 Ubiquitination of Petunia hybrida S3R and its mutant forms by self‐ (PhS3S3 ) pollen‐tube extracts (PTE). Fig. S13 Reduced seed set per capsule from T0 transgenic lines with mutated region (R) II of Petunia hybrida S3‐RNase. Fig. S14 Reduced seed set per capsule from T0 transgenic lines with mutated lysine or threonine within the six ubiquitinated residues of Petunia hybrida S3‐RNase. Fig. S15 Mutant (M) III largely maintains the biochemical and physical properties of Petunia hybrida S3‐RNase. Fig. S16Petuniahybrida S3‐RNase with mutated region (R) III markedly reduces cross seed sets. Fig. S17 Largely unaltered physicochemical properties of Petunia hybrida S3‐RNase with mutated region (R) I, II and III. Fig. S18 Reduced seed se [file NPH-231-1249-s001.pdf]

New Phytologist

New Phytologist Supporting Information

Article title: Primary restriction of S-RNase cytotoxicity by a stepwise ubiquitination and degradation pathway in *Petunia hybrida*

Authors: Hong Zhao, Yanzhai Song, Junhui Li, Yue Zhang, Huaqiu Huang, Qun Li, Yu'e Zhang, Yongbiao Xue

Article acceptance date: 20 April 2021

The following Supporting Information is available for this article:

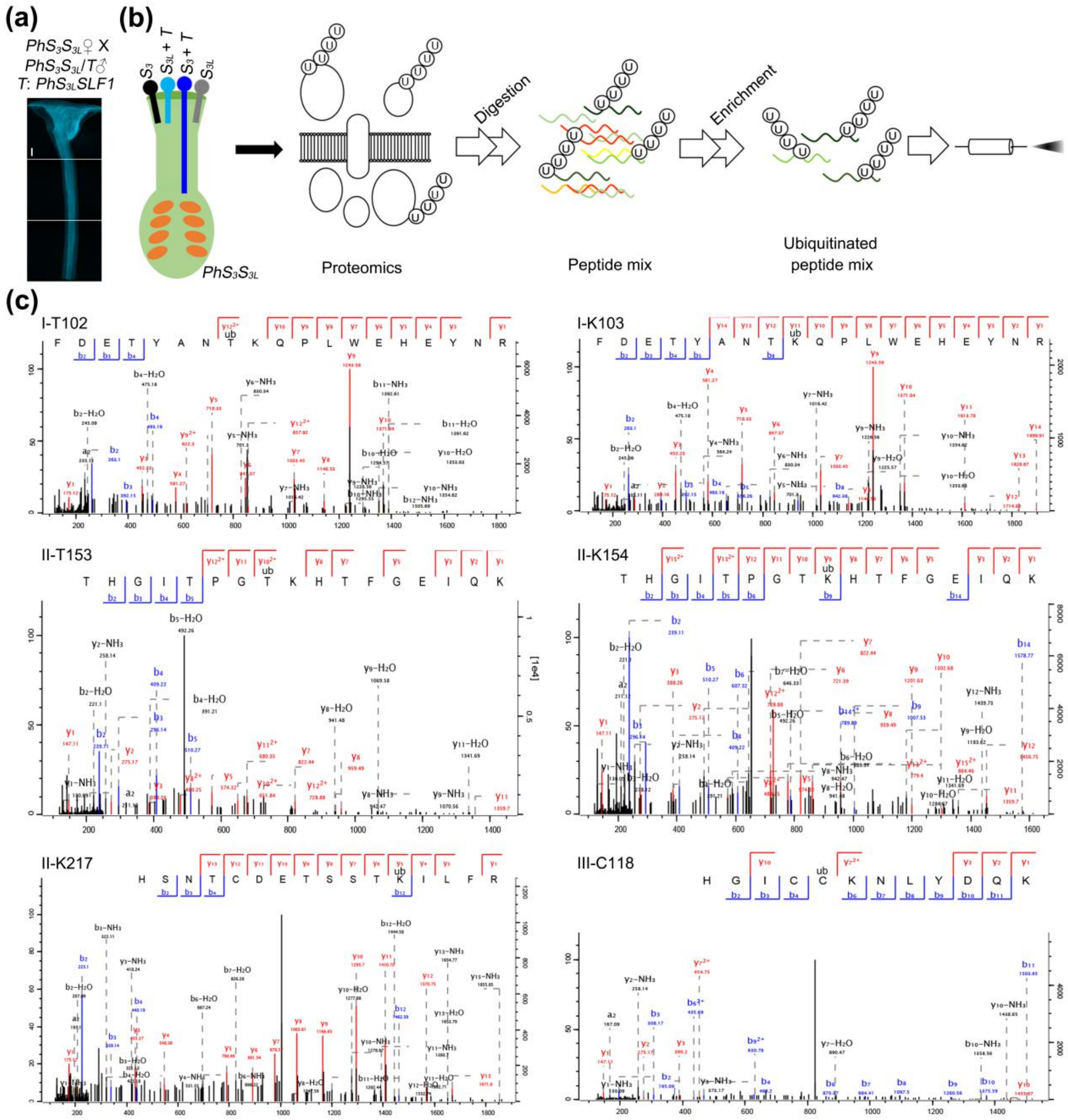

Fig. S1. Identification of six ubiquitinated residues of *Petunia hybrida* S$_3$-RNase by LC-MS/MS analysis of cross-pollinated pistils. (a) Aniline blue staining of pollen tubes in a wild-type (PhS_3S_{3L}) pistil cross-pollinated with transgenic pollen containing $PhS_{3L}SLF1$ ($PhS_3S_{3L}/PhS_{3L}SLF1$). Bar: 1 mm. **(b)** A flow chart of LC-MS/MS. PhS_3S_{3L} pistils pollinated with cross pollen of $PhS_3S_{3L}/PhS_{3L}SLF1$ containing transgenic $PhS_{3L}SLF1$ (T) are collected for protein extraction, digestion, ubiquitinated peptide enrichment and LC-MS/MS. **(c)** Tandem mass spectrometry (MS/MS) of ubiquitinated PhS$_3$-RNase peptide. Horizontal and vertical axes represent m/z and signal strengths, respectively. I, II and III indicate three ubiquitinated regions of PhS$_3$-RNase. T, K and C indicate threonine, lysine and cysteine.

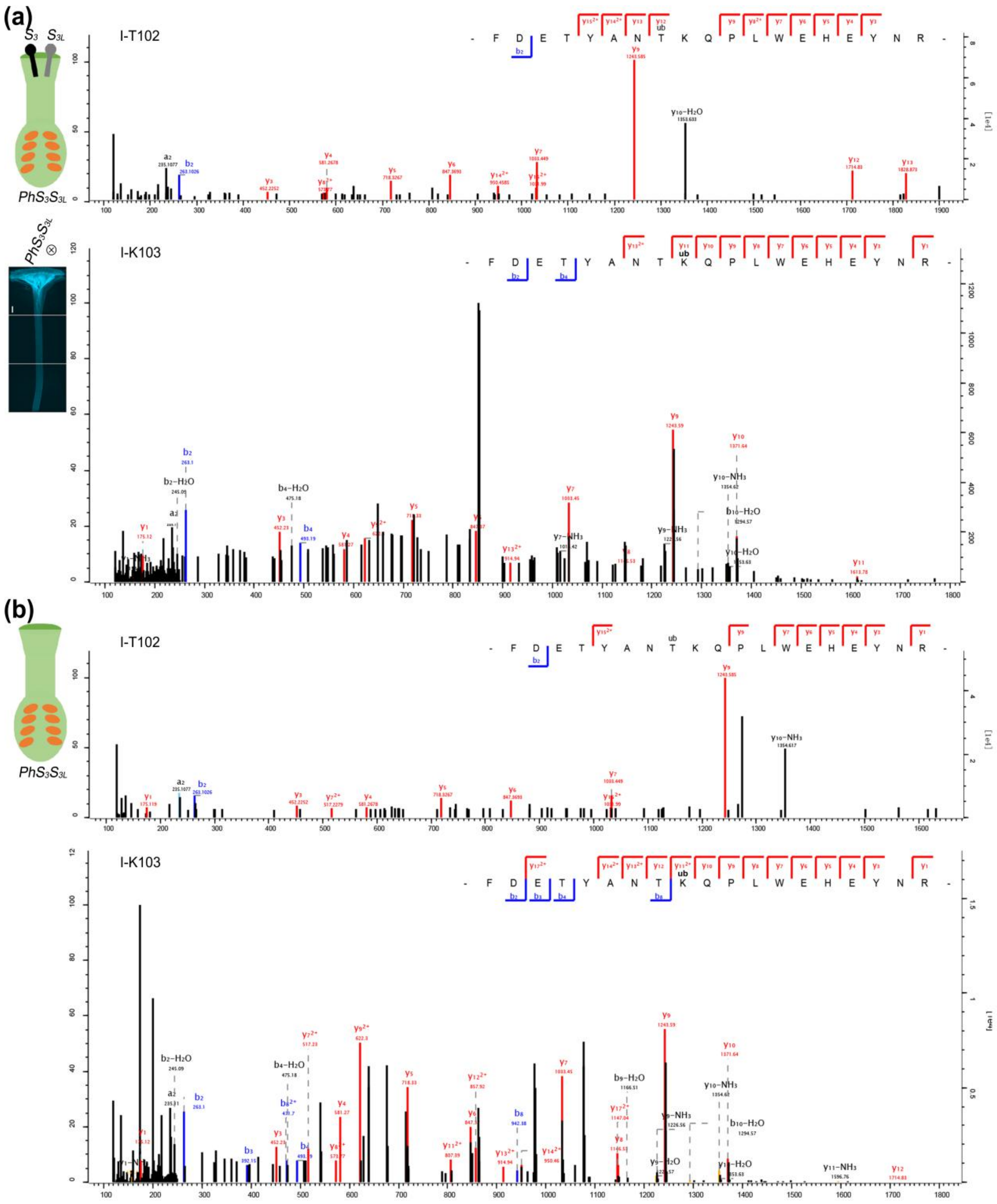

Fig. S2. Two ubiquitinated residues of *Petunia hybrida* S$_3$-RNase identified by LC-MS/MS analysis of self-pollinated and unpollinated pistils. (a) and **(b)** Tandem mass spectrometry (MS/MS) of ubiquitinated PhS$_3$-RNase peptide identified in self-pollinated **(a)** and unpollinated **(b)** pistils of wild type (*PhS$_3$S$_{3L}$*). Bar: 1 mm. T and K: threonine and lysine. Annotations of the horizontal and vertical axes of the MS/MS are identical to those of Fig. S1.

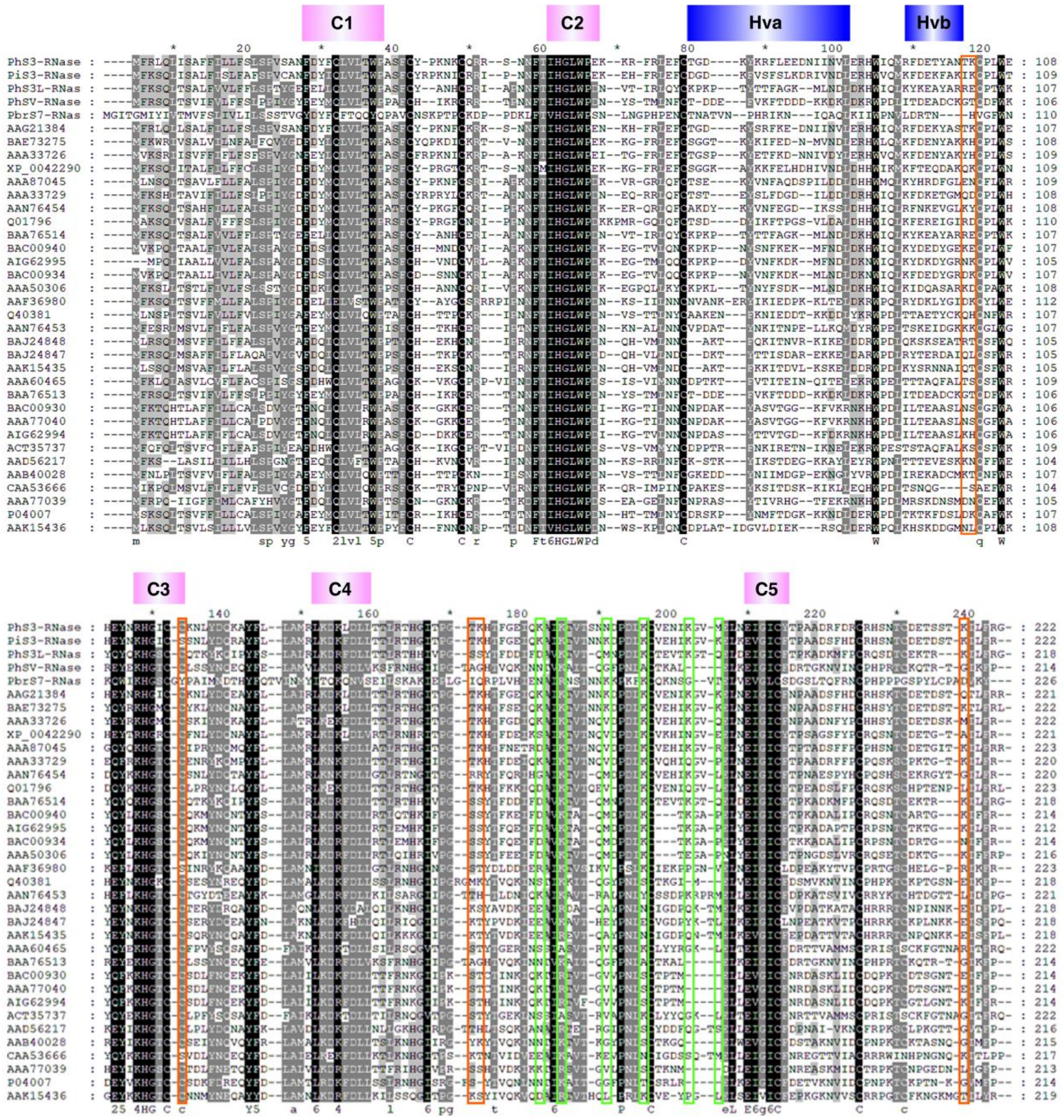

Fig. S3. Locations of six ubiquitinated residues of *Petunia hybrida* S₃-RNase in Solanaceous S-RNases. Amino acid sequence alignments of 36 S-RNases in Solanaceae. C1-C5, five conserved regions; Hva and Hvb, hypervariable region a and b. The black shadow indicates highly conserved sequences and the light less conserved ones. The orange rectangles indicate the ubiquitination sites of PhS$_3$-RNase, the green the major lysine residues identified previously (Hua and Kao, 2008) responsible for the ubiquitination and degradation of *Petunia inflata* S$_3$-RNase and their corresponding amino acids among the 36 S-RNases.

Hua, Z., and Kao, T.-H. 2008. Identification of major lysine residues of S$_3$-RNase of *Petunia inflata* involved in ubiquitin-26S proteasome-mediated degradation *in vitro*. *Plant Journal* **54**, 1094-1104.

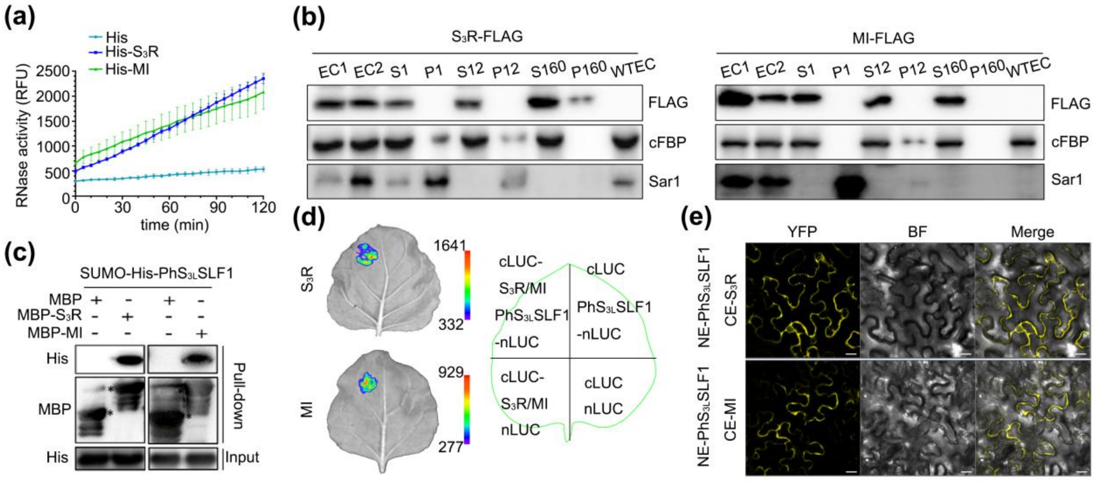

Fig. S4. ***Petunia hybrida*** **S$_3$-RNase with the mutated region (R) I displays largely unaltered biochemical and physical properties. (a)** RNase activity detection of His-S$_3$R and -mutant (M) I expressed by *pCold-TF* vectors. The relative fluorescence unit (RFU) indicating RNase activity during a time course experiment is shown as mean ± S.D. (n = 3). I: the ubiquitinated region I of PhS$_3$-RNase. **(b)** Immunoblot detection of FLAG-tagged S$_3$R and MI in subcellular fractions of *in vitro* germinated pollen tubes. EC1, EC2 and WTEC indicate entire cell homogenates of the pistils from the transgenic plants containing *S$_3$R-FLAG* or *MI-FLAG*, the pollen tubes of *PhS$_V$S$_V$* treated with EC1 and the pistils from wild-type *PhS$_3$S$_{3L}$*. WTEC was a negative control. S1 and P1, S12 and P12, S160 and P160 indicate supernatant and pellet fractions obtained by centrifugation of EC2 at 1,000 g, 12,000 g and 160,000 g, respectively. cFBP and Sar1 are marker antibodies of cytosol and endoplasmic reticulum (ER), respectively. **(c)** Physical interactions between PhS$_{3L}$SLF1 and S$_3$R and MI detected by pull-down assays. Input and pull-down: bait protein SUMO-His-PhS$_{3L}$SLF1 and prey proteins detected by immunoblots, respectively. MBP and SUMO-His are protein tags. Asterisks indicate bands of target proteins. **(d)** Split firefly luciferase complementation (SFLC) assay. The numbers on the left side of the color signal bars represent the values of the fluorescent signal. The injection positions of each component on tobacco leaves are indicated in the contour diagram of leaf margin. nLUC and cLUC: transiently expressed N-terminal and C-terminal regions of luciferase. **(e)** Bimolecular fluorescence complementation (BiFC) assays.

NE and CE: transiently expressed N-terminal and C-terminal regions of YFP by *pSPYNE* and *pSPYCE* vectors. YFP, BF and Merge represent the YFP fluorescence, bright field and their merged field, respectively. Bars: 20 μm.

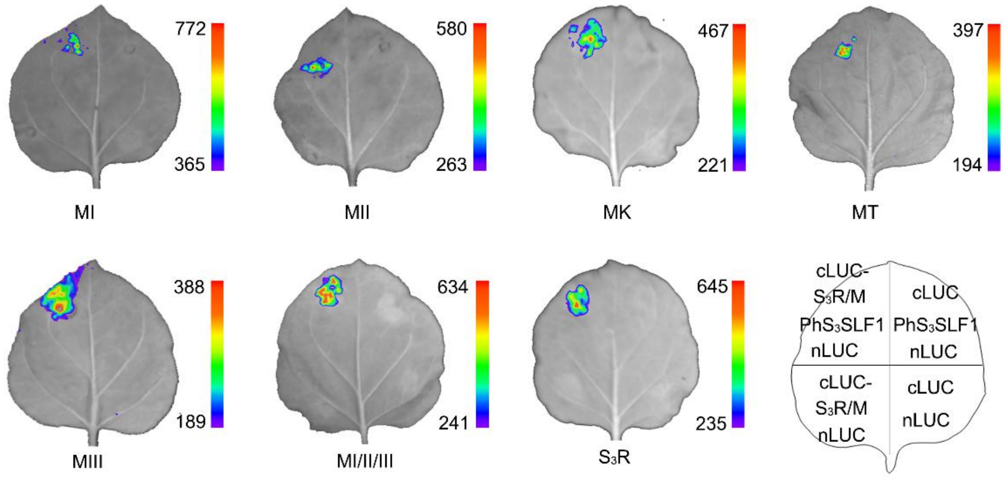

Fig. S5. Physical interactions between *Petunia hybrida* S_3R/Mutant (M) and PhS$_3$SLF1. S_3R/M represents wild-type or a mutant form of PhS$_3$-RNase. I, II and III indicate three ubiquitinated regions of PhS$_3$-RNase. K and T indicate lysine and threonine within six ubiquitinated residues of PhS$_3$-RNase. nLUC and cLUC indicate transiently expressed N-terminal and C-terminal regions of luciferase. The other annotations of this figure are identical to those of Fig. S4d.

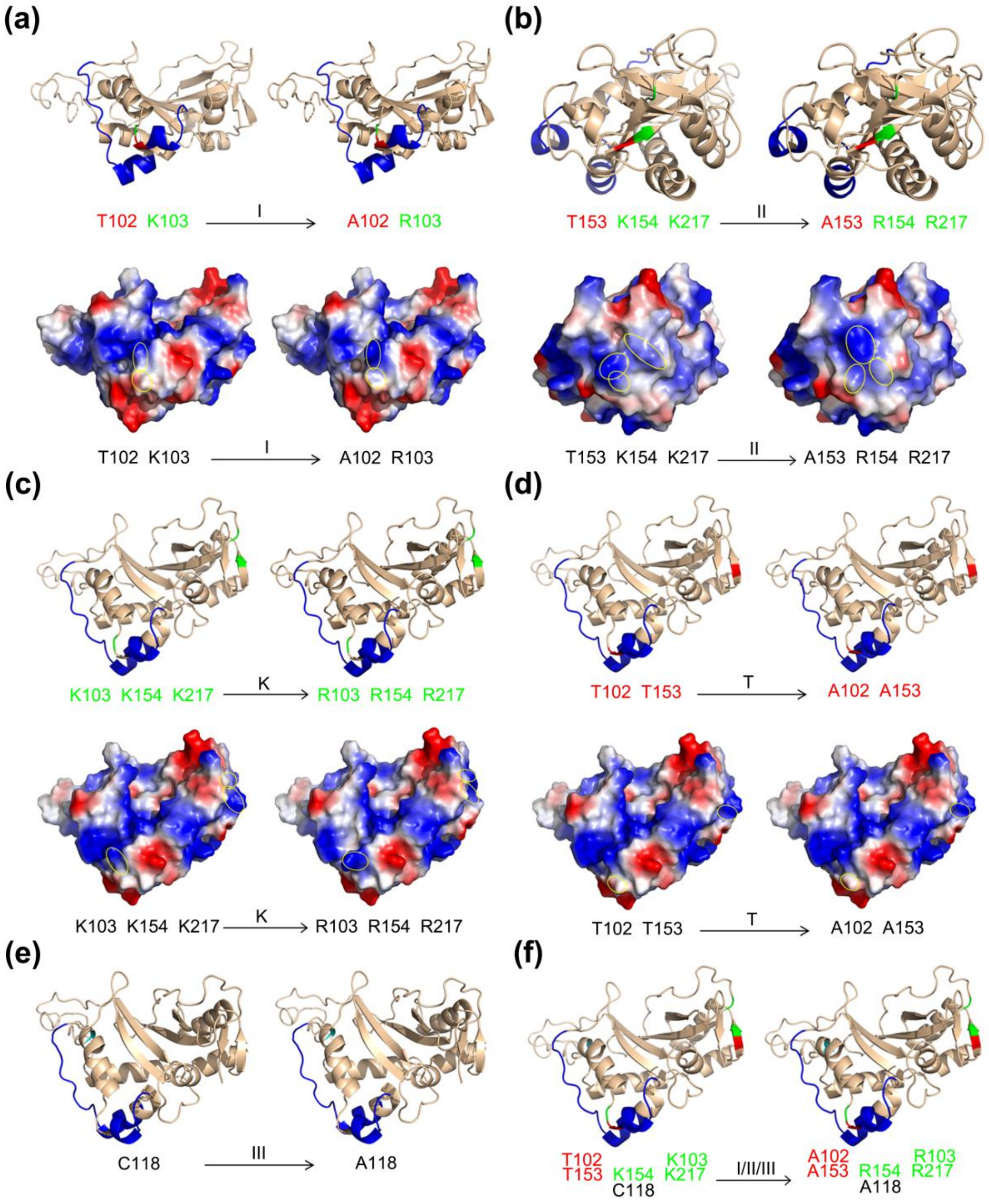

Fig. S6. Predicted three dimensional (3D) structures and surface electrostatic potentials of *Petunia hybrida* S$_3$-RNases with mutated ubiquitinated residues. **(a-d)** Predicted 3D structures (top) and surface electrostatic potentials (bottom) of PhS$_3$-RNase (left) and its mutant (M) forms (right). **(e,f)** Predicted 3D structures of PhS$_3$-RNase (left) and MIII (right) **(e)** or PhS$_3$-RNase (left) and MI/II/III (right) **(f)**. For the predicted 3D structures, blue indicates the hypervariable Hv regions, red T (threonine) and its mutant form A (alanine), and green K (lysine) and its mutant form R (arginine). For the predicated surface electrostatic potentials, blue and red indicate

positive and negative charges, respectively. I, II and III indicate three ubiquitinated regions of PhS_3-RNase.

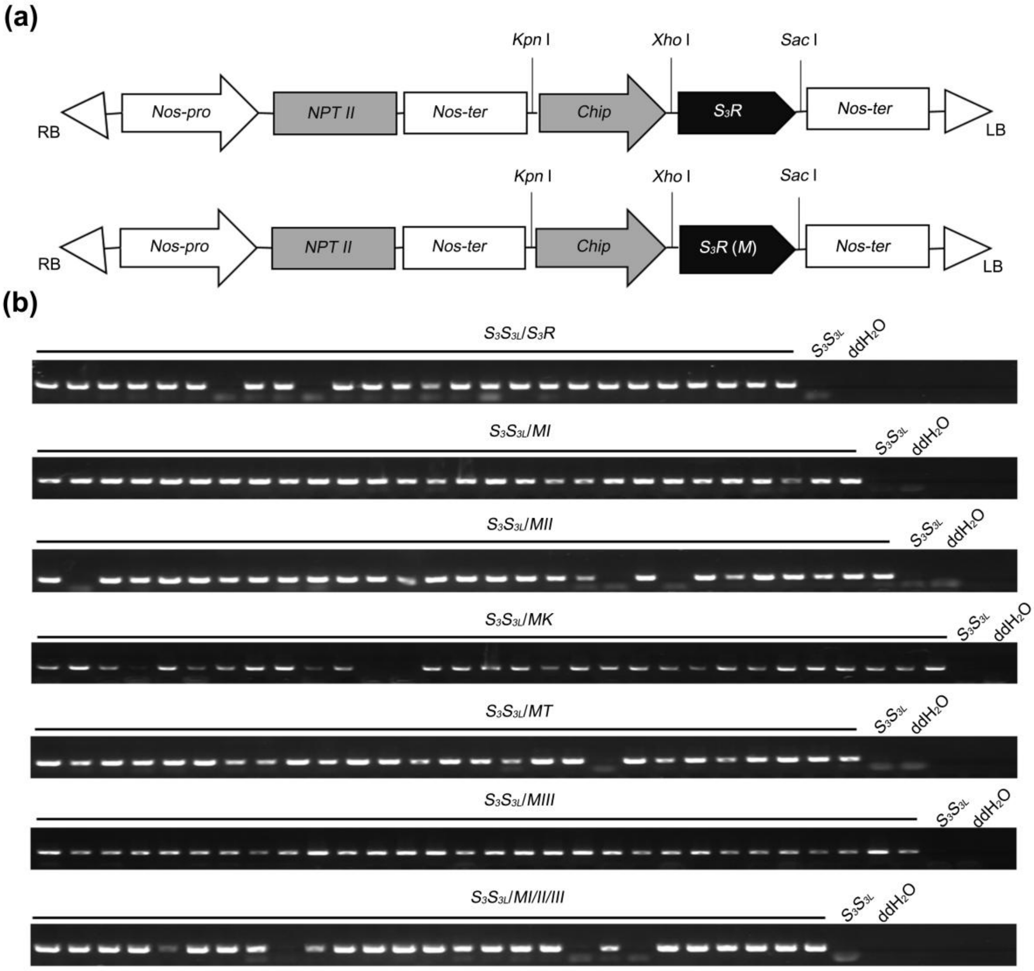

Fig. S7. ***Petunia hybrida*** ***S_3R*** **and** ***S_3R*** **(*Mutant*) (*M*) transgenes identification by PCR analysis. (a)** Schematic diagrams of plant expression constructs containing *S_3R* or *S_3R* (*M*) driven by a pistil-specific promoter *Chip* between right and left borders of T-DNA. *NPTII*, neomycin phospho-transferase II gene conferring kanamycin resistance. *Nos-pro* and *Nos-ter*, promoter and terminator of Nopaline synthase gene, respectively. *S_3R* (*M*) represents a mutant form of *S_3R*. **(b)** Identification of T_0 transgenic plants by genomic polymerase chain reaction (PCR). I, II and III: three ubiquitinated regions of PhS_3-RNase. K and T: lysine and threonine within six ubiquitinated residues of PhS_3-RNase. *S_3S_{3L}*: wild type and a negative control. H_2O: a negative control.

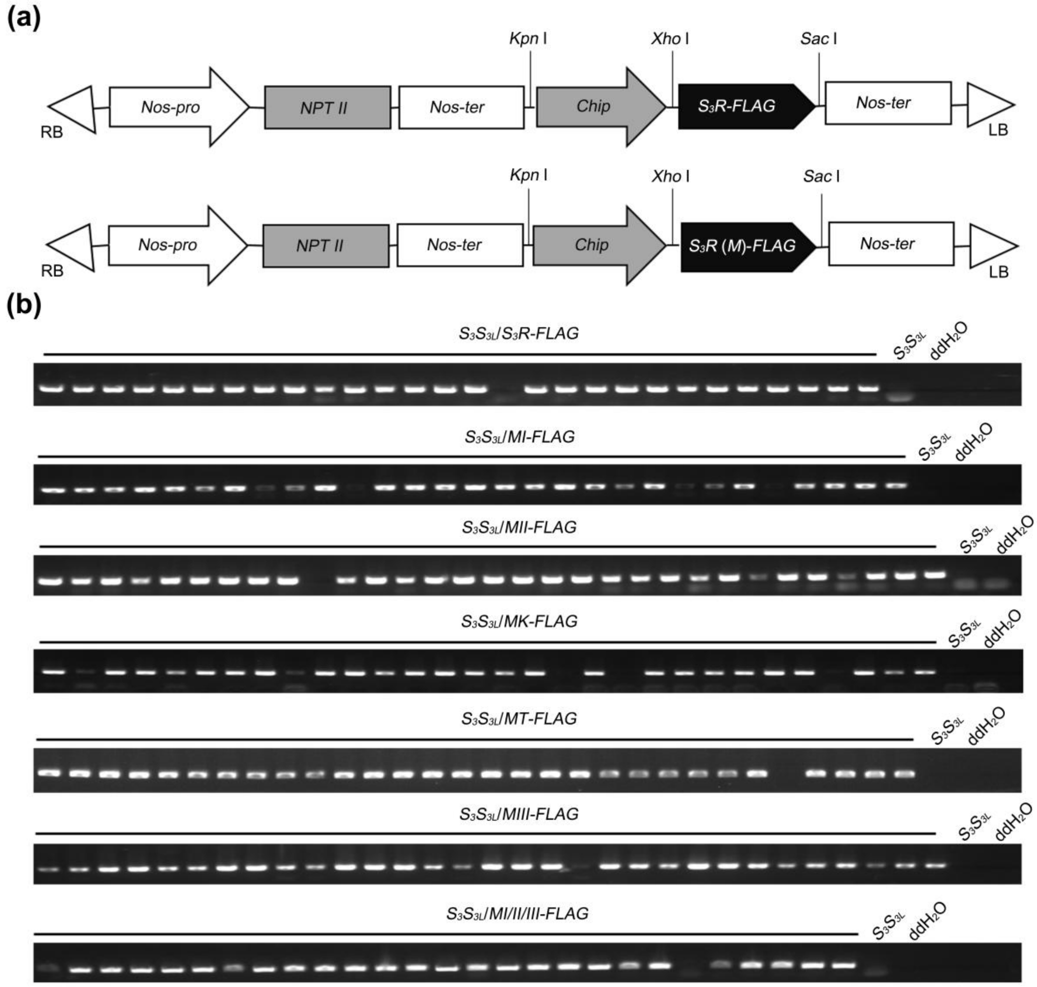

Fig. S8. Identification of *FLAG*-tagged *Petunia hybrida S_3R* and *S_3R* (*Mutant*) (*M*) transgenes by PCR analysis. (a) Schematic diagrams of plant expression constructs containing *S_3R-FLAG* or *S_3R* (*M*)-*FLAG*. *NPTII*, neomycin phospho-transferase II gene. *Nos-pro* and *Nos-ter*, promoter and terminator of Nopaline synthase gene. **(b)** Identification of T_0 transgenic plants by genomic PCR. I, II and III, three ubiquitinated regions of PhS3-RNase. K and T, lysine and threonine within six ubiquitinated residues of PhS3-RNase. Annotations of this figure are identical to those of Fig. S7.

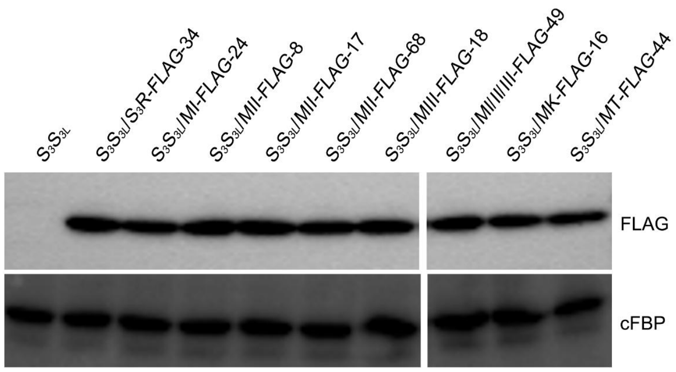

Fig. S9. Detection of *FLAG*-tagged *Petunia hybrida* S_3R and S_3R (*Mutant*) (*M*) transgenes expression by immunoblots. The genotypes and numbers of the transgenic lines are indicated on top. S_3S_{3L} is wild type used as a negative control. I, II and III: three ubiquitinated regions of PhS_3-RNase. K and T: lysine and threonine within six ubiquitinated residues of PhS_3-RNase. FLAG and cFBP antibodies were used to detect the transgene expression and sample loading, respectively.

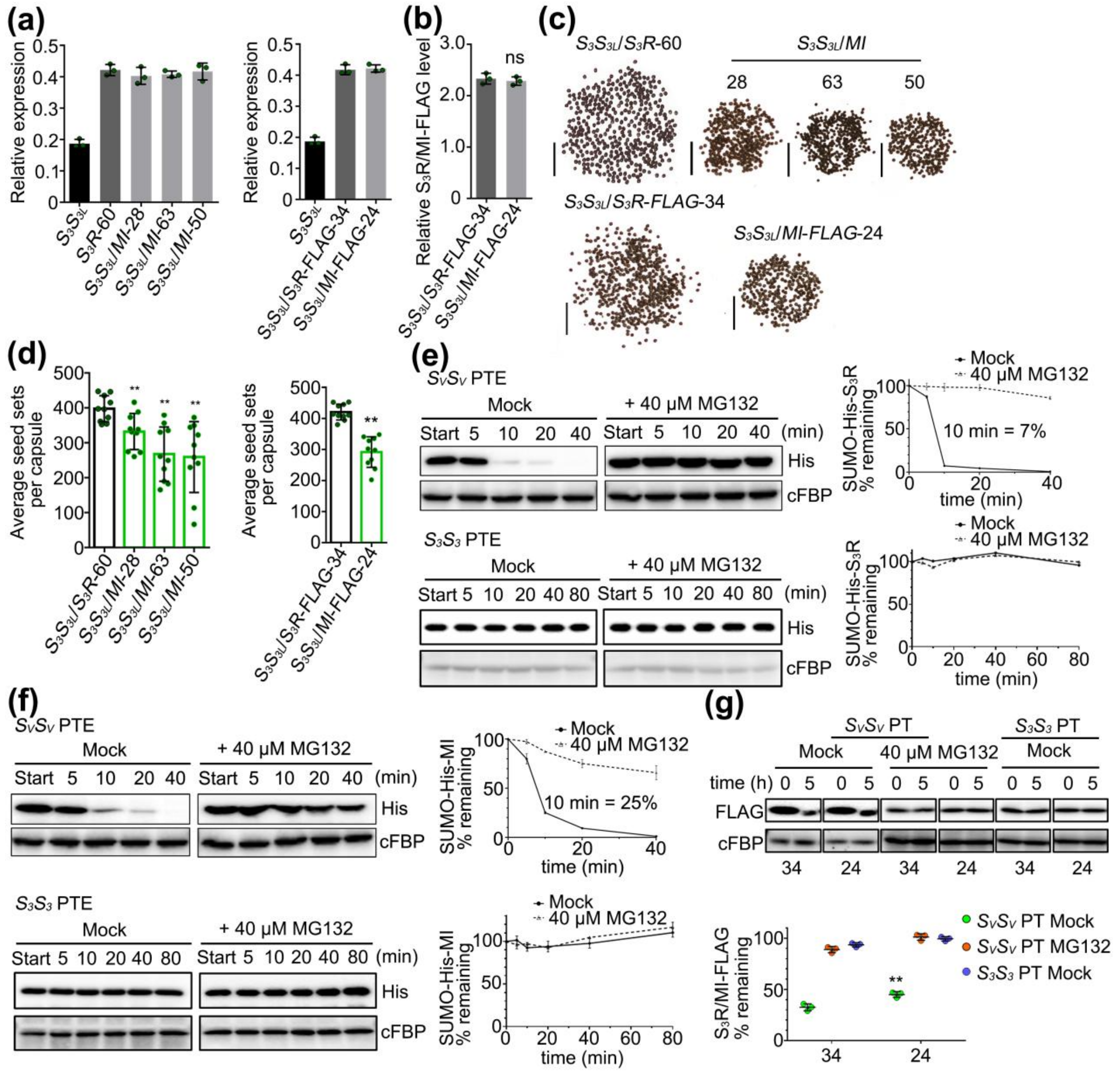

Fig. S10. *Petunia hybrida* S_3-RNase with the mutated region (R) I slightly reduces cross seed sets. (a) Transcripts of transgene (*PhS₃-RNase or mutant* (*M*) *I*) and native *PhS₃-RNase* detected by qRT-PCR. The T_0 transgenic lines are indicated below the horizontal axes. S_3S_{3L} is wild type. Data are shown as mean ± S.D. (n = 3). **(b)** Quantitative analyses of FLAG-tagged S_3R and MI proteins detected by immunoblots in Fig. S9. Data are shown as mean ± S.D. (n = 3). A student's *t*-test was used to generate the *p* values. ns (not significant), $p > 0.05$. **(c)** Reduced seed set per capsule from cross-pollinated T_0 transgenic lines containing *MI* or *MI-FLAG*. The transgenic plants containing *S_3R* or *MI* and *S_3R-FLAG* or *MII-FLAG* were pollinated with cross pollen of *PhS_VS_V*. Numbers below the horizontal lines indicate T_0 transgenic line numbers. Bars: 5 mm. **(d)** Statistical analyses of seed sets per

capsule from T_0 transgenic lines cross-pollinated with PhS_VS_V pollen. Data are shown as mean ± S.D. (n = 10). A student's *t*-test was used to generate the *p* values. **, $p < 0.01$. **(e)** and **(f)** Cell-free degradation of recombinant SUMO-His-tagged S_3R or MI by pollen-tube extracts (PTE) of PhS_VS_V (cross) or PhS_3S_3 (self). Left, immunoblots of the reaction products incubated with or without MG132 (Mock). Start, time point zero in each degradation assay. cFBP antibody was used to detect non-degraded loading control. Right, quantitative analyses of the degradation rates. Data are shown as mean ± S.D. (n = 3). The remaining amount at 10 min is indicated. **(g)** Time-course analyses of PhS_3R- and MI-FLAG levels in the cross- (PhS_VS_V) or self- (PhS_3S_3) pollen tubes (PT) incubated with or without MG132 (Mock). Top, immunoblots of PhS_3R- or MI-FLAG in the PT using FLAG antibody. cFBP was detected as a loading control. The numbers at the bottom indicate the transgenic line numbers corresponding to those in **d**. Bottom, quantitative analyses of the immunoblots. Data are shown as mean ± S.D. (n = 3). A student's *t*-test was used to generate the *p* values. **, $p < 0.01$.

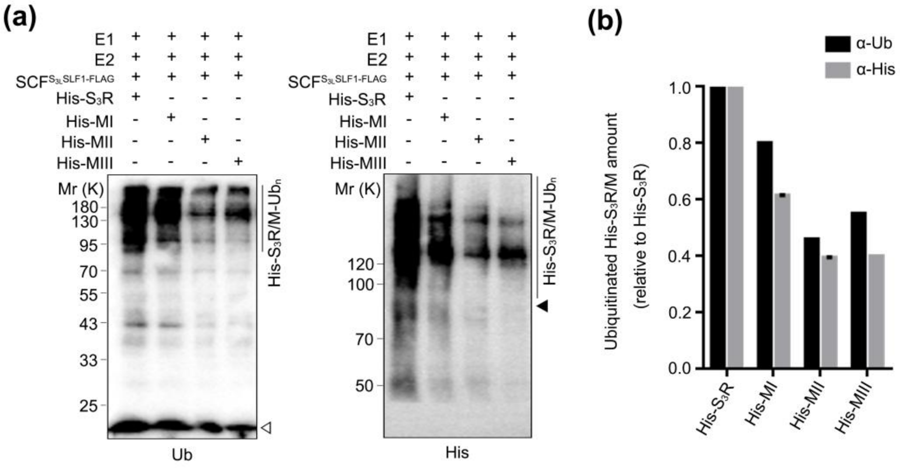

Fig. S11. Decreased ubiquitination amount of mutant (M) I, II and III mediated by SCF$^{S_{3L}SLF1}$ of *Petunia hybrida*. (a) Immunoblot detection of *in vitro* ubiquitinated recombinant His-tagged S$_3$R and its mutant forms using anti-ubiquitin (Ub) and -His antibodies. His-S$_3$R, -MI, -MII and -MIII are labeled as the substrates. I, II and III: three ubiquitinated regions of PhS$_3$-RNase. FLAG: a protein tag. PhS$_3$R/M indicates wild-type or a mutant form of PhS$_3$-RNase. The vertical lines illustrate the ubiquitinated His-S$_3$R/M. Open and filled arrowheads indicate ubiquitin and unubiquitinated His-S$_3$R/M monomers, respectively. **(b)** Quantitative analyses of immunoblots from **a**. Data are shown as mean ± S.D. (n = 3).

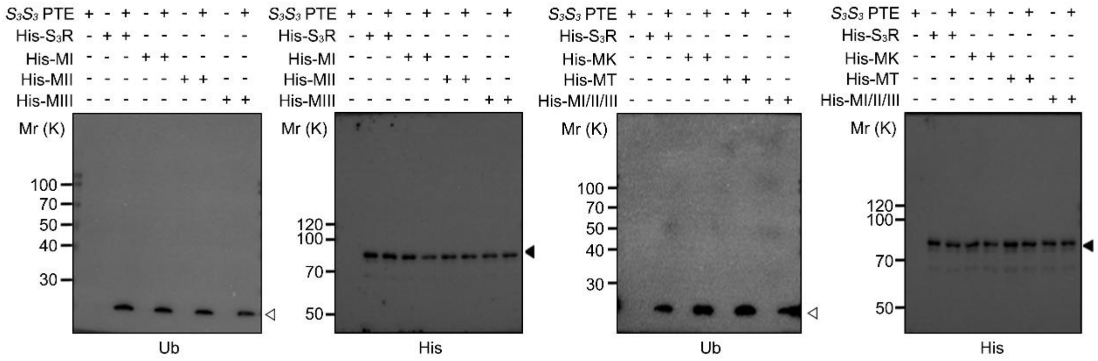

Fig. S12. Ubiquitination of *Petunia hybrida* S_3R and its mutant forms by self- (*Ph*S_3S_3) pollen-tube extracts (PTE). Immunoblots of cell-free ubiquitination products of His-tagged S_3R and its mutant (M) forms incubated with self- (*Ph*S_3S_3) PTE using anti-ubiquitin (Ub) and -His antibodies. I, II and III: three ubiquitinated regions of PhS_3-RNase. K and T: lysine and threonine within six ubiquitinated residues of PhS_3-RNase. Annotations of this figure are identical to those of Fig. S11a.

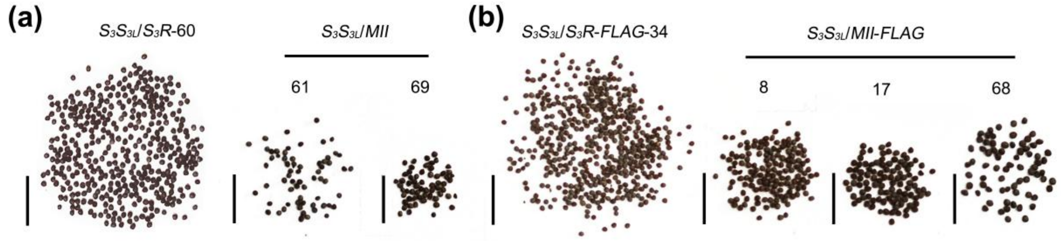

Fig. S13. Reduced seed set per capsule from T_0 transgenic lines with mutated region (R) II of *Petunia hybrida* S_3-RNase. The transgenic plants containing *S_3R* or *Mutant* (*M*) *II* **(a)** and *S_3R-FLAG* or *MII-FLAG* **(b)** were pollinated with cross pollen of *PhS_VS_V*. Numbers below the horizontal lines are T_0 transgenic line numbers. FLAG: a protein tag. Bars: 5 mm.

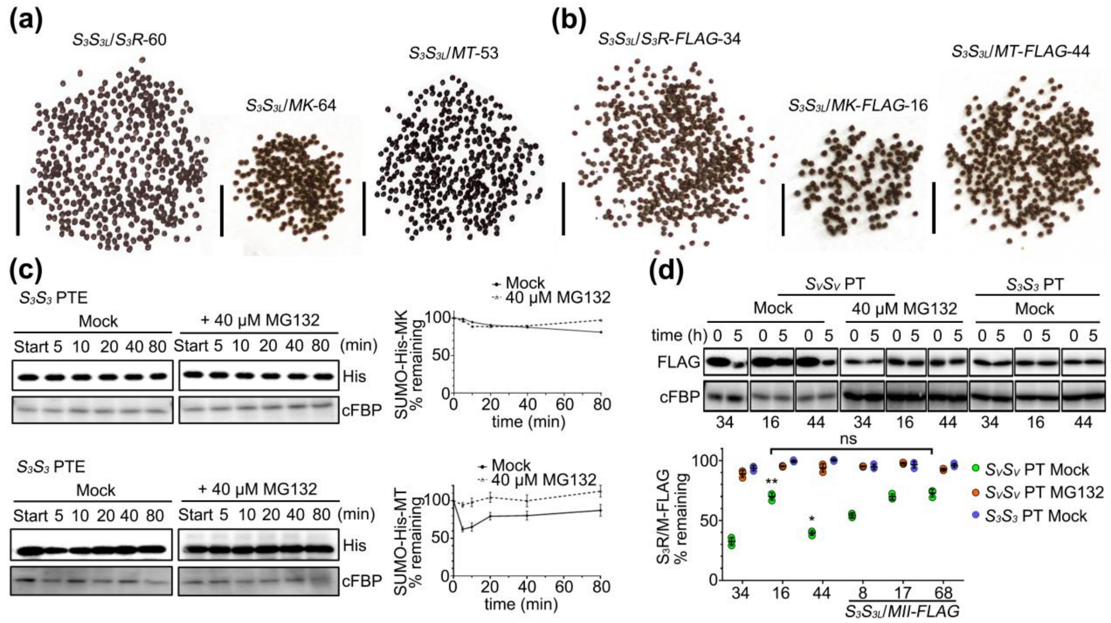

Fig. S14. Reduced seed set per capsule from T_0 transgenic lines with mutated lysine or threonine within the six ubiquitinated residues of *Petunia hybrida* S_3-RNase. (a) and **(b)** The transgenic plants containing *S_3R*, *mutant* (*M*) *K* or *MT* **(a)** and *S_3R*-, *MK*- or *MT-FLAG* **(b)** were pollinated with cross pollen of *PhS_VS_V*. FLAG: a protein tag. Bars: 5 mm. **(c)** Cell-free degradation of recombinant SUMO-His-MK, and -MT by self (*PhS_3S_3*) pollen-tube extracts (PTE). Data are shown as mean ± S.D. (n = 3). **(d)** Time-course analyses of MK- and MT-FLAG levels in the cross- (*PhS_VS_V*) or self- (*PhS_3S_3*) pollen tubes (PT) incubated with or without MG132. The numbers at the bottom of the immunoblots and the x-axis indicate the transgenic line numbers corresponding to those in **b**. Data are shown as mean ± S.D. (n = 3). A student's *t*-test was used to generate the *p* values. *, $p < 0.05$; **, $p < 0.01$; ns (not significant), $p > 0.05$. Annotations of this figure are identical to those of Fig. S10.

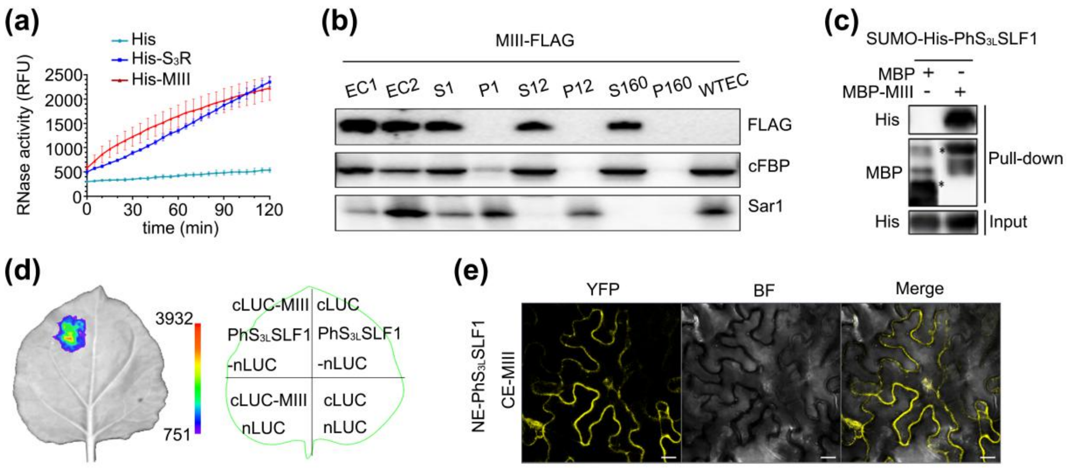

Fig. S15. Mutant (M) III largely maintains the biochemical and physical properties of *Petunia hybrida* S$_3$-RNase. (a) RNase activity detection of His-S$_3$R and -MIII expressed by *pCold-TF* vectors. Data are shown as mean ± S.D. (n = 3). RFU: relative fluorescence unit. III: the ubiquitinated region III of PhS$_3$-RNase. **(b)** Immunoblot detection of FLAG-tagged MIII in subcellular fractions of *in vitro* germinated pollen tubes. EC1, EC2 and WTEC indicate entire cell homogenates of the pistils from the transgenic plants containing *MIII-FLAG*, the pollen tubes of *PhS$_V$S$_V$* treated with EC1 and the pistils from wild-type *PhS$_3$S$_{3L}$*. S1 and P1, S12 and P12, S160 and P160 indicate respective supernatant and pellet fractions obtained by centrifugation of EC2 at 1,000 g, 12,000 g and 160,000 g. **(c)** The physical interaction between PhS$_{3L}$SLF1 and MIII detected by pull-down assay. MBP and SUMO-His are protein tags. **(d)** SFLC assay. nLUC and cLUC indicate transiently expressed N-terminal and C-terminal regions of luciferase. **(e)** BiFC assay. NE and CE: transiently expressed N-terminal and C-terminal regions of YFP. YFP, BF and Merge: YFP fluorescence, bright field and their merged field, respectively. Bars: 20 µm. Annotations of this figure are identical to those of Fig. S4.

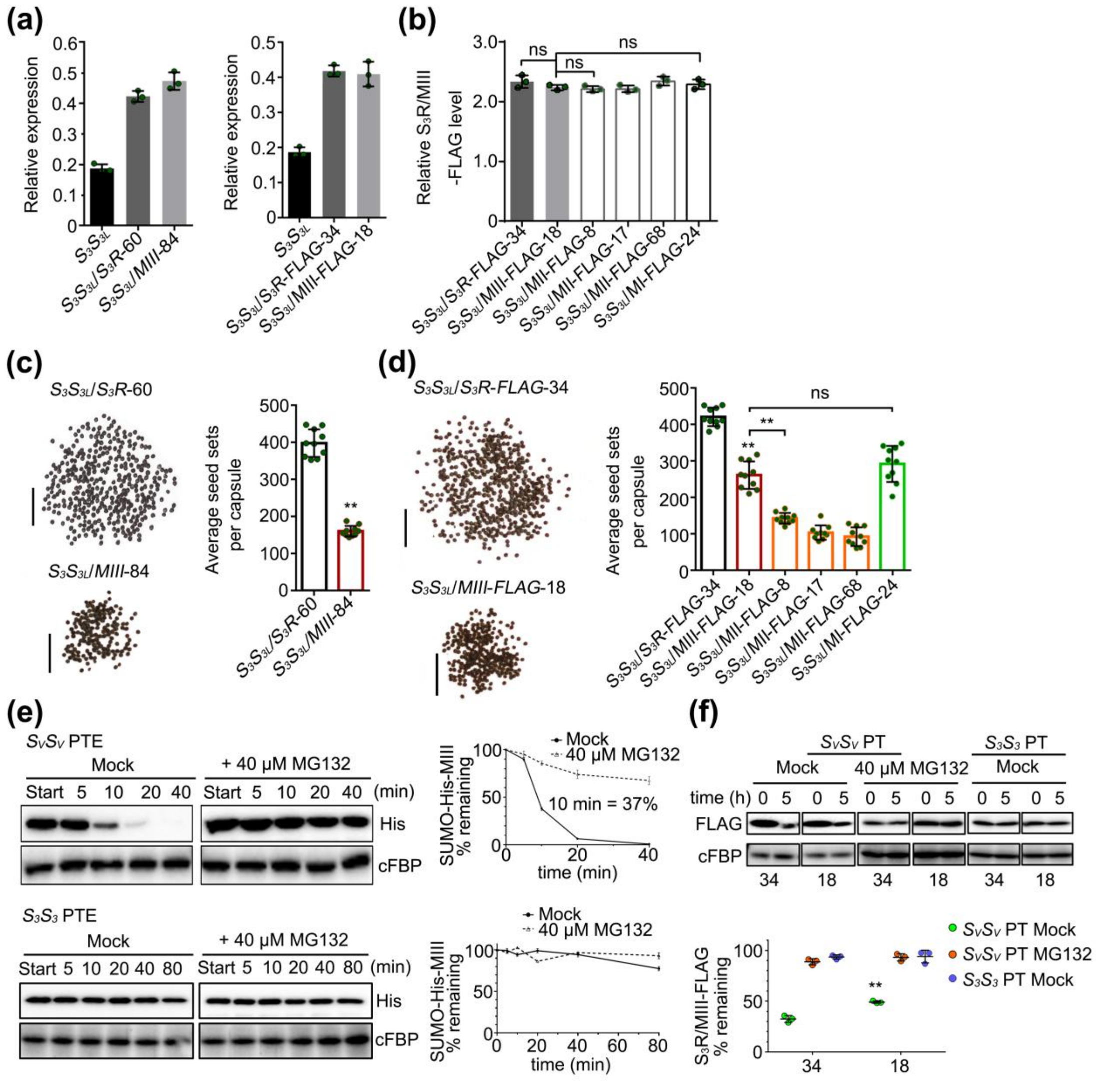

Fig. S16. ***Petunia hybrida*** **S_3-RNase with mutated region (R) III markedly reduces cross seed sets.** **(a)** Transcripts of transgene (*PhS$_3$-RNase or mutant* (*M*) *III*) and native *PhS$_3$-RNase* detected by qRT-PCR. Data are shown as mean ± S.D. (n = 3). FLAG, a protein tag. **(b)** Quantitative analyses of S_3R- and MIII-FLAG proteins detected by immunoblots in Fig. S9. Data are shown as mean ± S.D. (n = 3). Student's *t*-test: ns (not significant), $p > 0.05$. **(c)** Reduced seed sets per capsule from cross-pollinated T_0 transgenic lines containing *MIII* and their statistical analyses. Data are shown as mean ± S.D. (n = 10). Student's *t*-test: **, $p < 0.01$. Bars: 5 mm. **(d)** Reduced seed sets per capsule from cross-pollinated T_0 transgenic lines containing *MIII-FLAG* and their statistical analyses. Data are shown as mean ± S.D. (n = 10). Student's *t*-test: ns (not significant), $p > 0.05$; **, $p < 0.01$. Bars: 5 mm. **(e)**

Left: immunoblots of recombinant SUMO-His-MIII in the cell-free degradation products incubated with or without MG132 (Mock). PTE: pollen-tube extracts. Right: quantitative analyses of the immunoblots. Data are shown as mean ± S.D. (n = 3). (**f**) Top: immunoblots of MIII-FLAG levels in the cross- (PhS_VS_V) or self- (PhS_3S_3) pollen tubes (PT) incubated with or without MG132. Bottom: quantitative analyses of the immunoblots. The x-axis represents the transgenic line numbers corresponding to those in **d**. Data are shown as mean ± S.D. (n = 3). Student's *t*-test: **, $p < 0.01$. Annotations of this figure are identical to those of Fig. S10.

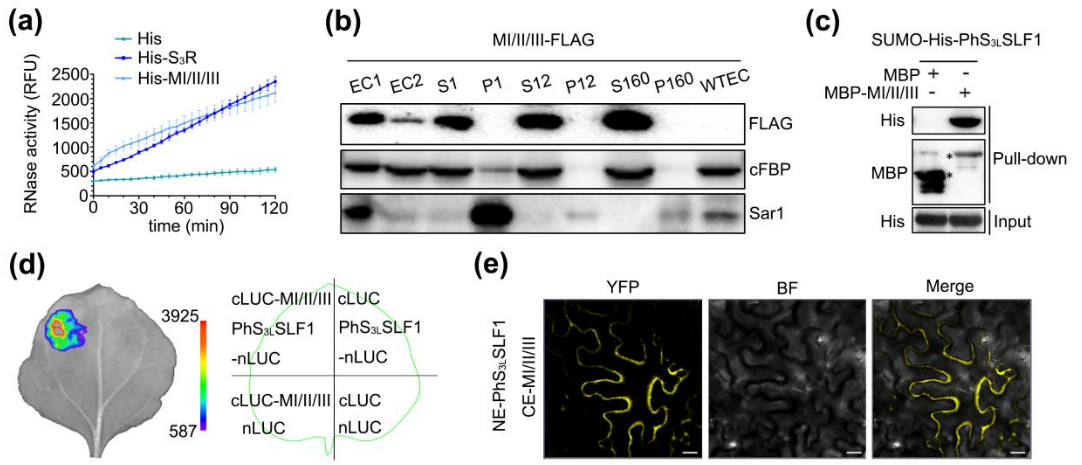

Fig. S17. Largely unaltered physicochemical properties of *Petunia hybrida* S_3-RNase with mutated region (R) I, II and III. (a) RNase activity detection of His-Mutant (M) I/II/III expressed by *pCold-TF* vectors. RFU: relative fluorescence unit. Data are shown as mean ± S.D. (n = 3). **(b)** Immunoblot detection of FLAG-tagged MI/II/III in subcellular fractions of *in vitro* germinated pollen tubes. EC1, EC2 and WTEC indicate entire cell homogenates of the pistils from the transgenic plants containing *MI/II/III-FLAG*, the pollen tubes of *PhS$_V$S$_V$* treated with EC1 and the pistils from wild-type *PhS$_3$S$_{3L}$*. S1 and P1, S12 and P12, S160 and P160 indicate supernatant and pellet fractions obtained by centrifugation of EC2 at 1,000 g, 12,000 g and 160,000 g, respectively. **(c)** The physical interaction between PhS$_{3L}$SLF1 and MI/II/III detected by pull-down assay. MBP and SUMO-His are protein tags. **(d)** SFLC assay. nLUC and cLUC: transiently expressed N-terminal and C-terminal regions of luciferase. **(e)** BiFC assay. NE and CE: transiently expressed N-terminal and C-terminal regions of YFP. YFP, BF and Merge: YFP fluorescence, bright field and their merged field. Bars: 20 μm. Annotations of this figure are identical to those of Fig. S4.

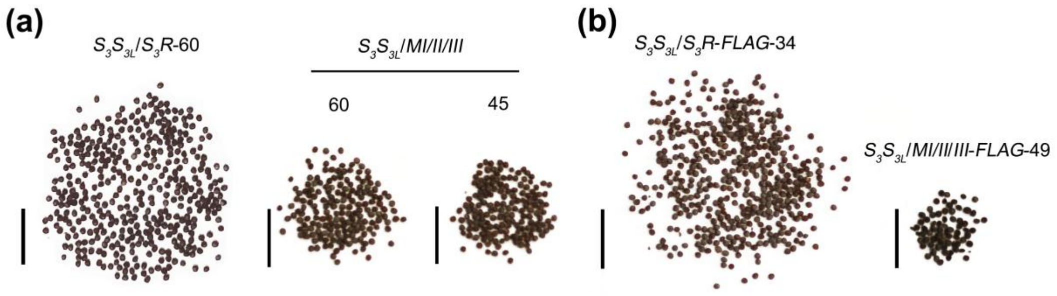

Fig. S18. Reduced seed set per capsule from T_0 transgenic lines with mutated region (R) I/II/III of *Petunia hybrida* S_3-RNase. The transgenic plants containing *mutant* (*M*) *I*/*II*/*III* **(a)** or *MI*/*II*/*III-FLAG* **(b)** were pollinated with cross pollen of *PhS$_V$S$_V$*. Numbers below the horizontal lines indicate T_0 transgenic line numbers. FLAG: a protein tag. Bars: 5 mm.

Table S1. List of primer sequences.

Primers	Sequences (5' to 3')	Purposes
Chip-F	ggtaccCTAGCTAGAAGATCTCTTTTGT	P1
Chip-R	ctcgagCCTGGAGATCTAATTTTCTTTT	P1
S_3R-F	ctcgagATGTTTAGATTACAGCTCATAT	P1
S_3R-R	gagctcTCAACCCCGAAACAGAATCTT	P1
S_3R-*FLAG*-R	gagctcCTACTTGTCATCGTCGTCCTTG<br>TAATCACCCCGAAACAGAATC	P1
S_3R-*K217R*-R	gagctcTCAACCCCGAAACAGAATCTCT	P1
S_3R-*K217R-FLAG*-R	gagctcCTACTTGTCATCGTCGTCCTTG<br>TAATCACCCCGAAACAGAATTC	P1
T102AK103R-F	TGACGAAACATACGCCAACGCC<br>AGACAACCTCTCTGGGAGC	P2
T102AK103R-R	TCTGGCGTTGGCGTATGTTTCGT<br>CAAACCTCATTTGAATCCA	P2
T153AK154R-F	TCATGGAATTACTCCGGGAGCCA<br>GACATACATTTGGTGAAAT	P2
T153AK154R-R	TCTGGCTCCCGGAGTAATTCCAT<br>GAGTTCTGAGAGTTGTCAA	P2
T102A-F	TGACGAAACATACGCCAACGCCA<br>AGCAACCTCTCTGGGA	P2
T102A-R	GGCGTTGGCGTATGTTTCGTCAA<br>ACCTCATTTGAATCCA	P2
K103R-F	AAACATACGCCAACACTAGACAA<br>CCTCTCTGGGAGCACG	P2
K103R-R	TCTAGTGTTGGCGTATGTTTCGTC<br>AAACCTCATTTGAAT	P2
T153A-F	TCATGGAATTACTCCGGGAGCCA<br>AGCATACATTTGGTGA	P2

Table S1 (Continued 1)

Primers	Sequences (5' to 3')	Purposes
T153A-R	GGCTCCCGGAGTAATTCCATGAGTTCTGAGAGTTGTCAA	P2
K154R-F	GAATTACTCCGGGAACAAGACATACATTTGGTGAAATCC	P2
K154R-R	TCTTGTTCCCGGAGTAATTCCATGAGTTCTGAGAGTTGT	P2
C118A-F	CAATAGGCATGGAATTTGCGCCAAAAATCTCTACGATCA	P2
C118A-R	GGCGCAAATTCCATGCCTATTGTATTCGTGCTCCCAGAG	P2
18S rRNA-F	CGGCTACCACATCCAAGGAA	P3
18S rRNA-R	TGTCACTACCTCCCCGTGTCA	P3
S_3R-F1	TCGTCTTAACATGGCCAGCA	P3
S_3R-R1	CCAGTGGCGCTCTAGAACAT	P3
S_3R-F2	ACCTCTCTGGGAGCACGAAT	P3
S_3R-R2	TCAAACCTATCTGCCGCTGG	P3
S_3R-F3	ACTCCGGGAACAAAGCATACA	P3
S_3R-R3	AGAATCTTCGTGCTGCTCGT	P3
S_3R-F	gaattcAGTGCGAATTTTGACTACTTCC	P4
S_3R-R	gtcgacTCAACCCCGAAACAGAATCTTC	P4
S_3R-*K217R*-R	gtcgacTCAACCCCGAAACAGAATCTCT	P4
S_3R-F	GGGGACAAGTTTGTACAAAAAAGCAGCTCCAGTGCGAATTTTGACTACTTCC	P5
S_3R-R	GGGGACCACTTTGTACAAGAAAGCTGGGTCACCCCGAAACAGAATCTTCGTG	P5
S_3R-*K217R*-R	GGGGACCACTTTGTACAAGAAAGCTGGGTCACCCCGAAACAGAATTCTCGTG	P5

Table S1 (Continued 2)

Primers	Sequences (5' to 3')	Purposes
$S_{3L}SLF1$-F	GGGGACAAGTTTGTACAAAAAAGCAG<br>CTCCATGGCGAATGGTATTTTAAAGA	P5
$S_{3L}SLF1$-R	GGGGACCACTTTGTACAAGAAAGCTG<br>GGTCAAATTTTTGTACTTTTGTACTG	P5
S_3R-F	ggatccAGTGCGAATTTTGACTACTTCC	P6
S_3R-R	ctcgagTCAACCCCGAAACAGAATCTTC	P6
S_3R-K217R-R	ctcgagTCAACCCCGAAACAGAATTCT	P6
$S_{3L}SLF1$-F	ggatccATGGCGAATGGTATTTTAAAGA	P6
$S_{3L}SLF1$-R	ctcgagCTAAAATTTTTGTACTTTTGTA	P6
S_3R-F	GGGGACAAGTTTGTACAAAAAAGCAG<br>GCTCCAGTGCGAATTTTGACTACTTCC	P7
S_3R-R	GGGGACCACTTTGTACAAGAAAGCTG<br>GGTCACCCCGAAACAGAATCTTCGTG	P7
S_3R-K217R-R	GGGGACCACTTTGTACAAGAAAGCTG<br>GGTCACCCCGAAACAGAATTCTCGTG	P7

F: forward primer; R: reverse primer; P1: *pBI101* construction; P2: *Petunia hybrida* *S_3R* mutagenesis; *T* and *A*: threonine and its mutant form alanine; *K* and *R*: lysine and its mutant form arginine; P3: qRT-PCR; P4: *pMAL-c2x* construction; P5: BiFC or SFLC; P6: *pET30a* construction; P7: *pCold-TF* construction.

Table S2. Names and accession numbers of S-RNases used in this study.

Names	Accession numbers [a]
Petunia hybrida S_3-RNase	U07363
Petunia hybrida S_{3L}-RNase	AJ271065
Petunia hybrida S_V-RNase	AJ271062
Pyrus bretschneideri S_7-RNase	XM_009350009
Petunia inflata S_2-RNase	AAG21384
Petunia inflata S_3-RNase	AAA33727
Petunia inflata S_{k1}-RNase	BAE73275
Petunia inflata S_1-RNase	AAA33726
Solanum lycopersicum S_3-RNase	XP_004229063
Nicotiana alata S_{A2}-RNase	AAA87045
Petunia hybrida S_X-RNase	AAA33729
Petunia axillaris S_{C2}-RNase	AAN76454
Solanum tuberosum S_2-RNase	Q01796
Petunia hybrida S_{B2}-RNase	BAA76514
Solanum neorickii S-RNase	BAC00940
Solanum habrochaites S_2-RNase	AIG62995
Solanum chilense S_1-RNase	BAC00934
Solanum chacoense S_{11}-RNase	AAA50306
Solanum chacoense S_{14}-RNase	AAF36980
Nicotiana alata S_7-RNase	Q40381
Petunia axillaris S_{C1}-RNase	AAN76453
Petunia hybrida S_{11}-RNase	BAJ24848
Petunia hybrida S_7-RNase	BAJ24847
Petunia axillaris S_1-RNase	AAK15435
Solanum habrochaites S_4-RNase	AIG62997
Petunia hybrida S_1-RNase	AAA60465
Petunia hybrida S_{B1}-RNase	BAA76513

Table S2 (Continued)

Names	Accession numbers [a]
Solanum peruvianum S_{22}-RNase	BAC00930
Solanum peruvianum S_{12}-RNase	AAA77040
Solanum habrochaites S_1-RNase	AIG62994
Petunia hybrida S_0-RNase	ACT35737
Solanum chacoense S_{12}-RNase	AAD56217
Nicotiana alata S_6-RNase	AAB40028
Solanum peruvianum S_3-RNase	CAA53666
Solanum peruvianum S_{11}-RNase	AAA77039
Nicotiana alata S_2-RNase	P04007
Petunia axillaris S_{13}-RNase	AAK15436

(a) Accession numbers are from National Center for Biotechnology Information (NCBI) protein database (https://www.ncbi.nlm.nih.gov/).

Table S3. Seed sets of S_3S_{3L}/S_3R and S_3S_{3L}/S_3R (*Mutant*) (*M*) T_0 transgenic plants of *Petunia hybrida.*

T_0 transgenic lines [a]	Genotype of pollen donor		Seed set [d]
	S_3S_{3L} [b] (Self)	S_VS_V [c] (Cross)	
S_3S_{3L}/S_3R-60	0/5	10/10	398 ± 37
S_3S_{3L}/MI-28	0/5	10/10	332 ± 51
S_3S_{3L}/MI-63	0/5	10/10	268 ± 78
S_3S_{3L}/MI-50	0/5	10/10	259 ± 101
S_3S_{3L}/MII-61	0/5	10/10	89 ± 19
S_3S_{3L}/MII-69	0/5	9/10	59 ± 6
S_3S_{3L}/MK-64	0/5	10/10	207 ± 28
S_3S_{3L}/MT-53	0/5	10/10	356 ± 16
$S_3S_{3L}/MIII$-84	0/5	10/10	160 ± 14
$S_3S_{3L}/MI/II/III$-60	0/5	10/10	207 ± 32
$S_3S_{3L}/MI/II/III$-45	0/5	10/10	186 ± 17

(a) Numbers of T_0 transgenic plants containing S_3R and its mutant forms (wild type/transgene-line number). I, II and III indicate three ubiquitinated regions of PhS3-RNase. K and T indicate lysine and threonine within six ubiquitinated residues of PhS3-RNase. **(b)** Numbers represent those of mature capsules/pistils pollinated by self pollen of S_3S_{3L}. **(c)** Numbers represent those of mature capsules/pistils pollinated by cross pollen of S_VS_V. **(d)** Data are shown as average seed set per capsule ± S.D., n ≥ 9.

Table S4. Seed sets of *S_3S_{3L}/S_3R-FLAG* and *S_3S_{3L}/S_3R* (*Mutant*) (*M*)-*FLAG* T_0 transgenic plants of *Petunia hybrida*.

T_0 transgenic lines [(a)]	Genotype of pollen donor		Seed sets [(d)]
	S_3S_{3L} [(b)] (Self)	S_VS_V [(c)] (Cross)	
S_3S_{3L}/S_3R-FLAG-34	0/5	10/10	421 ± 25
S_3S_{3L}/MI-FLAG-24	0/5	10/10	292 ± 49
S_3S_{3L}/MII-FLAG-8	0/5	10/10	143 ± 14
S_3S_{3L}/MII-FLAG-17	0/5	10/10	104 ± 19
S_3S_{3L}/MII-FLAG-68	0/5	10/10	93 ± 25
S_3S_{3L}/MK-FLAG-16	0/5	10/10	113 ± 17
S_3S_{3L}/MT-FLAG-44	0/5	10/10	334 ± 16
S_3S_{3L}/MIII-FLAG-18	0/5	10/10	261 ± 37
S_3S_{3L}/MI/II/III-FLAG-49	0/5	10/10	93 ± 34

(a) Numbers of T_0 transgenic plants containing *FLAG*-tagged *S_3R* and its mutant forms (wild type/transgene-line number). **(b, c)** Annotations are identical to those of Table S3. **(d)** Data are shown as average seed set per capsule ± S.D., n = 10.
